# A novel group decision making method based on CoCoSo and interval-valued Q-rung orthopair fuzzy sets

**DOI:** 10.1038/s41598-024-56922-5

**Published:** 2024-03-19

**Authors:** Yan Zheng, Hongwu Qin, Xiuqin Ma

**Affiliations:** 1 College of Computer Science and Engineering, Northwest Normal University, Lanzhou, 730070 Gansu China; 2 Institute for Big Data Analytics and Artificial Intelligence (IBDAAI), Universiti Teknologi MARA, 40450 Shah Alam, Selangor Malaysia

**Keywords:** Interval-valued Q-rung orthopair fuzzy sets, MAGDM, CoCoSo, Sepsis diagnose, Applied mathematics, Computational science, Computer science, Information technology, Pure mathematics, Scientific data, Software, Statistics

## Abstract

Interval-valued q-rung orthopair fuzzy set (IVq-ROFS) is a powerful tool for dealing with uncertainty. In this paper, we first propose a new method for aggregating multiple IVq-ROFSs, which is easier to understand and implement in the multi-attribute group decision making process compared to current aggregation operators. Secondly, this paper introduces a new fuzzy entropy with parameters based on IVq-ROFS, which is highly flexible due to its adjustable parameters. Based on this, the IVq-ROFS-based attribute weight calculation method is proposed to obtain the objective weights of the attributes, which is more reasonable and objective than the existing methods. Then, for the dimensional differences between the three compromise scores in the original Combined Compromise Solution (CoCoSo) method, the enhanced compromise scores are proposed. These scores are obtained by normalizing the three dependent compromise scores, ensuring that they fall within the same range. Finally, a novel CoCoSo mothed on IVq-ROFS using the proposed fuzzy entropy and enhanced compromise scores is presented. The proposed method is highly adaptable and scalable, not limited to IVq-ROFS. The excellent performance and robustness of the proposed method are verified in sepsis diagnosis applications.

## Introduction

The fuzziness and imprecision of the data in many real-world problems make it extremely difficult to make effective data-driven decisions^1–3^. Numerous mathematical models have been developed in recent decades to deal with uncertainties. Zadeh^4^ proposed the concept of fuzzy sets as a generalization of crisp sets. Takeuti^5^ extended the traditional fuzzy set theory and introduced the concept of intuitionistic fuzzy sets (IFS). IFSs introduced the non-membership degree (NMD) as the supplement to the membership degree (MD), which has been extensively applied in many fields, including decision making^6,7^, cluster analysis^8,9^, medical diagnosis^10,11^, and pattern recognition^12,13^. Turksen^14^ proposed the concept of interval-valued fuzzy set. Atanassov^15^ further extended IFSs and proposed the concept of interval valued intuitionistic fuzzy set (IVIFS) to make the consideration more comprehensive in practical applications. IVIFSs use subintervals in the interval [0,1] to describe the MD and NMD, making them more flexible and practical than IFSs when dealing with ambiguous or uncertain data. Yager^16^ proposed Pythagorean fuzzy set (PFS), which extended the concept of IFS and provided new research ideas and methods for solving uncertainty problems. This theory is more widely used in the field of MADM^17^. Compared with IFS, PFS have the sum of squares of MD and NMD, which are less than or equal to 1, allowing decision makers to give a wider range of values. Later, Zhang^18^ further extended PFS and proposed the concept of Interval-valued Pythagorean Fuzzy Set (IVPFS). Furthermore, in order to cover more data, fermatean fuzzy sets^19,20^ and interval-valued fermatean fuzzy sets^21^ were presented. Integration of IFS, PFS and fermatean fuzzy sets described above, q-rung orthopair fuzzy^22–25^ set was proposed. When the q-ROFS will degenerate to IFS, PFS, FFS. Similarly, interval valued q-rung orthopair fuzzy set (IVq-ROFS)^22^ was proposed, and when the IVq-ROFS will degenerate to IVIFS, IVPFS, IVFFS. Compared with IVIFS, IVPFS, IVFFS, and IVq-ROFS have more data that can be covered than IVIFS, IVPFS, and IVFFS. Based on the above models, there are many extension models such as picture fuzzy set^26,27^ , complex fuzzy set, fuzzy soft set^28^ and their extensions^29–37^. The current research on IVq-ROFS mainly focuses on the aggregation operators^38–40^, information measures^41–43^, extended IVq-ROFS^44–46^, MADM methods^47–49^.

Experts and scholars have studied IVq-ROFS and its applications in MADM from different perspectives and proposed many valuable decision-making methods. The q-connection number was shown in^50,51^, and a new MAGDM method was proposed based on the q-connection number and IVq-ROFS. A scientific risk evaluation method based on IVq-ROFS capable of solving these deficiencies in the existing method was given in^51^. Seker et al.^52^ proposed a COPRAS method for IVq-ROFS. Yang et al.^53^ focused on the preference relations of IVq-ROFS and presented a related GDM method. Gurmani et al.^54^ created a new approach to determining expert weights using distance and similarity measures for IVq-ROFS.

However, there are some research gaps below. From a theoretical point of view, firstly, for MAGDM for IVq-ROFS, it is necessary to aggregate information from many different experts. However, the existing multi-source information aggregation method in^55^, which used the Archimedean Muirhead mean operators to aggregate fuzzy data, is not easy to understand and implement. Secondly, the existing method in^52,55–57^ related to attribute weight determination has a certain degree of subjectivity. Therefore, presenting a weight calculation method with complete objectivity is essential. Thirdly, for the original CoCoSo method^58^ proposed by Yazidani et al., which combined three comprised scores to calculate the final rating, there is an unavoidable problem that the value domains of the three dependent compromise scores are different, which directly affects the final score calculation. It is necessary to convert three dependent compromise scores to a unified scale for the CoCoSo method. From a practical point of view, in diagnosing disease, the diagnostic process in clinical care cannot rely solely on test data, as there is often excessive variability in patients' signs and symptoms and a lack of specificity. If a patient with suspected or confirmed infection presents with a worsening condition and is at risk of organ dysfunction, early recognition and prompt treatment as appropriate is critical to improving outcomes. By the time the diagnosis becomes apparent, and multiple physiologic parameters are abnormal, the risk of death is very high. Therefore, multi-expert collective decision making is crucial in clinical diagnosis because MAGDM can gather the decision-making ideas of multiple experts and finally unify them to complete the initial diagnosis of the disease at an early stage when the patient has fewer signs and data abnormalities. The rate of misdiagnosis of the conventional clinical diagnosis may be higher, doctors are in a state of uncertainty about the diagnosis of sepsis, the interval- value q-rung fuzzy MAGDM can be a good solution of this problem.

The main contributions of this paper are as follows:

1. A novel multi-source aggregation method of aggregating multiple IVq-ROFSs from various specialists is introduced. Compared to the traditional method, it is easier to understand and implement.
2. A novel fuzzy entropy with parameter derived from IVq-ROFS, which is more flexible and versatile compared to conventional fuzzy entropy. Additionally, the IVq-ROFS-based attribute weight calculation method is proposed, utilizing this fuzzy entropy as a basis.
3. This work aims to enhance the dimensional distinctions among the three compromise scores of the original CoCoSo approach.
4. There is a limited number of fuzzy MAGDM methods available for sepsis diagnosis. Therefore, we want to validate the accuracy and applicability of our proposed method using a case study on sepsis diagnosis.

The structure of this paper is as follows: “Preliminaries” section describes the related concepts of IVq-ROFS and the original CoCoSo method. In “A novel CoCoSo group decision making method on IVq-ROFSs” section defines the multi-source information aggregation method, presents the new fuzzy entropy based on IVq-ROFS and the proposed CoCoSo group decision method. In “Case Study: Sepsis diagnosis” section describes the application of our method in Sepsis dignose case. In “Sensitivity analysis and comparative analysis” section, we make sensitivity analysis and the comparative analysis with other methods to verify feasibility of our method. And then the merits of our method are discussed. In “Conclusion” section we conclude this paper.

## Preliminaries

This section introduces the definitions associated with interval-valued q-rung orthopair fuzzy sets. It then describes the fundamental principles of fuzzy entropy for existing interval-valued fuzzy sets. Finally, the algorithmic flow of the original CoCoSo technique is presented.

### Interval-valued fuzzy set

#### Definition 1

^14^ Interval-valued fuzzy set (IVFS): Let the universe X be a non-empty set, then the IVFS on X can be expressed as:

$$A=\left\{\left(x,\left[{\mu }_{A}^{-}\left(x\right),{\mu }_{A}^{+}\left(x\right)\right]|x\in X\right)\right\},$$

where ${\mu }_{A}^{-}\left(x\right)$ is the lower bound of MD, ${\mu }_{A}^{+}\left(x\right)$ is the upper bound of MD.

### Interval-valued intuitionistic fuzzy set

#### Definition 2

^15^ Interval-valued intuitionistic fuzzy set(IVIFS):Let the universe X be a non-empty set, then the IVIFS on X can be expressed as:

$${A}^{I*}=\left\{\langle x,{\mu }_{{A}^{I*}}\left(x\right),{\nu }_{{A}^{I*}}\left(x\right)\rangle |x\in X\right\},$$

where ${\mu }_{{A}^{I*}}\left(x\right)\in \left({\mu }_{{A}^{I*}}^{-},{\mu }_{{A}^{I*}}^{+}\right)$, ${\nu }_{{A}^{I*}}\left(x\right)\in \left({\nu }_{{A}^{I*}}^{-},{\nu }_{{A}^{I*}}^{+}\right)$ and $0\le {\mu }_{{A}^{I*}}^{-}\le {\mu }_{{A}^{I*}}^{+}\le 1$, $0\le {\nu }_{{A}^{I*}}^{-}\le {\nu }_{{A}^{I*}}^{+}\le 1$. ${\mu }_{{A}^{I*}}\left(x\right):X\to \left[\mathrm{0,1}\right]$ and ${\nu }_{{A}^{I*}}\left(x\right):X\to \left[\mathrm{0,1}\right]$ for all $x\in X$ meet ${\mu }_{{A}^{I*}}^{+}+{\nu }_{{A}^{I*}}^{+}\le 1$. ${\mu }_{{A}^{I*}}\left(x\right)$ and ${\nu }_{{A}^{I*}}\left(x\right)$ denote the MD and NMD of elements in $X$ belonging to ${A}^{I*}$ ,respectively.

### Interval-valued pythagorean fuzzy set

#### Definition 3

^18^ Interval-valued Pythagorean fuzzy set (IVPFS):Let the universe X be a non-empty set, then the IVPFS on X can be expressed as:

$${A}^{P*}=\left\{\langle x,{\mu }_{{A}^{P*}}\left(x\right),{\nu }_{{A}^{P*}}\left(x\right)\rangle |x\in X\right\},$$

where ${\mu }_{{A}^{P*}}\left(x\right)\in \left({\mu }_{{A}^{P*}}^{-},{\mu }_{{A}^{P*}}^{+}\right)$, ${\nu }_{{A}^{P*}}\left(x\right)\in \left({\nu }_{{A}^{P*}}^{-},{\nu }_{{A}^{P*}}^{+}\right)$ and $0\le {\mu }_{{A}^{P*}}^{-}\le {\mu }_{{A}^{P*}}^{+}\le 1$, $0\le {\nu }_{{A}^{P*}}^{-}\le {\nu }_{{A}^{P*}}^{+}\le 1$. ${\mu }_{{A}^{P*}}\left(x\right):X\to \left[\mathrm{0,1}\right]$ and ${\nu }_{{A}^{P*}}\left(x\right):X\to \left[\mathrm{0,1}\right]$ for all $x\in X$ meet ${\left({\mu }_{{A}^{P*}}^{+}\right)}^{2}+{\left({\nu }_{{A}^{P*}}^{+}\right)}^{2}\le 1$. ${\mu }_{{A}^{P*}}\left(x\right)$ and ${\nu }_{{A}^{P*}}\left(x\right)$ denote the MD and NMD of elements in $X$ belonging to ${A}^{P*}$, respectively.

### Interval-valued Fermatean fuzzy set

#### Definition 4

^21^ Interval-valued Fermatean fuzzy set (IVFFS): Let the universe X be a non-empty set, then the IVFFS on X can be expressed as:

$${A}^{F*}=\left\{\langle x,{\mu }_{{A}^{F*}}\left(x\right),{\nu }_{{A}^{F*}}\left(x\right)\rangle |x\in X\right\},$$

where ${\mu }_{{A}^{F*}}\left(x\right)\in \left({\mu }_{{A}^{F*}}^{-},{\mu }_{{A}^{F*}}^{+}\right)$, ${\nu }_{{A}^{F*}}\left(x\right)\in \left({\nu }_{{A}^{F*}}^{-},{\nu }_{{A}^{F*}}^{+}\right)$ and $0\le {\mu }_{{A}^{F*}}^{-}\le {\mu }_{{A}^{F*}}^{+}\le 1$, $0\le {\nu }_{{A}^{F*}}^{-}\le {\nu }_{{A}^{F*}}^{+}\le 1$. ${\mu }_{{A}^{F*}}\left(x\right):X\to \left[\mathrm{0,1}\right]$ and ${\nu }_{{A}^{F*}}\left(x\right):X\to \left[\mathrm{0,1}\right]$ for all $x\in X$ meet ${\left({\mu }_{{A}^{F*}}^{+}\right)}^{3}+{\left({\nu }_{{A}^{F*}}^{+}\right)}^{3}\le 1$. ${\mu }_{{A}^{F*}}\left(x\right)$ and ${\nu }_{{A}^{F*}}\left(x\right)$ denote the MD and NMD of elements in $X$ belonging to ${A}^{F*}$, respectively.

### Interval-valued q-rung orthopair fuzzy set

#### Definition 5

^22^ Interval-valued q-rung orthopair fuzzy set (IVq-ROFS): Let the universe X be a non-empty set, then the IVq-ROFS on X can be expressed as:

**Figure 1:**
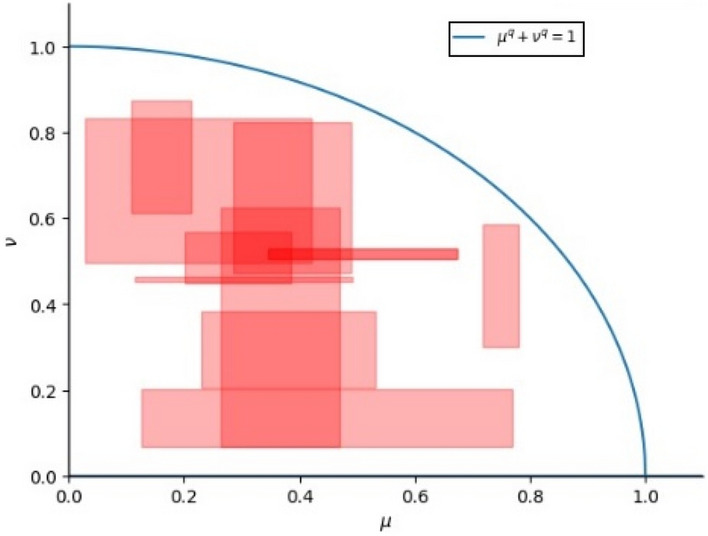
The values of MD and NMD of IVq-ROFS.

1 $$ \begin{array}{*{20}c} {A^{Q*} = \left\{ {x,\mu_{{A^{Q*} }} \left( x \right),\nu_{{A^{Q*} }} \left( x \right)|x \in X} \right\},} \\ \end{array} $$

where ${\mu }_{{A}^{Q*}}\left(x\right)\in \left({\mu }_{{A}^{Q*}}^{-},{\mu }_{{A}^{Q*}}^{+}\right)$, ${\nu }_{{A}^{Q*}}\left(x\right)\in \left({\nu }_{{A}^{Q*}}^{-},{\nu }_{{A}^{Q*}}^{+}\right)$ and $0\le {\mu }_{{A}^{Q*}}^{-}\le {\mu }_{{A}^{Q*}}^{+}\le 1$, $0\le {\nu }_{{A}^{Q*}}^{-}\le {\nu }_{{A}^{Q*}}^{+}\le 1$. ${\mu }_{{A}^{Q*}}\left(x\right):X\to \left[\mathrm{0,1}\right]$ and ${\nu }_{{A}^{Q*}}\left(x\right):X\to \left[\mathrm{0,1}\right]$ for all $x\in X$ meet ${\left({\mu }_{{A}^{Q*}}^{+}\right)}^{q}+{\left({\nu }_{{A}^{Q*}}^{+}\right)}^{q}\le 1$. ${\mu }_{{A}^{Q*}}\left(x\right)$ and ${\nu }_{{A}^{Q*}}\left(x\right)$ denote the MD and NMD of elements in $X$ belonging to ${A}^{Q*},$ respectively. The values of MD and NMD of IVq-ROFS are as shown in Fig. 1.

For each IVq-ROFS on ${X}^{Q*}$, the hesitancy degree in^20^ ${\pi }_{{A}^{Q*}}\left(x\right)\in \left({\pi }_{Q*}^{-},{\pi }_{Q*}^{+}\right)$ is given, where

2 $$\begin{array}{c}\left\{\begin{array}{l}{\pi }_{Q*}^{-}=\sqrt[q]{1-{\left({\mu }_{{A}^{Q*}}^{+}\right)}^{q}-{\left({\nu }_{{A}^{Q*}}^{+}\right)}^{q}}\\ {\pi }_{Q*}^{+}=\sqrt[q]{1-{\left({\mu }_{{A}^{Q*}}^{-}\right)}^{q}-{\left({\nu }_{{A}^{Q*}}^{-}\right)}^{q}}\\ \left({\pi }_{Q*}^{-},{\pi }_{Q*}^{+}\right)\in \left(\mathrm{0,1}\right)\end{array}\right.\end{array}$$

For IVq-ROFS the following basic operations are established.

3 $$\begin{aligned}{A}_{1}^{Q*}  ={A}_{2}^{Q*} & \Leftrightarrow \left[{\mu }_{{A}_{1}^{Q*}}\left(x\right),{\nu }_{{A}_{1}^{Q*}}\left(x\right)\right]=\left[{\mu }_{{A}_{2}^{Q*}}\left(x\right),{\nu }_{{A}_{2}^{Q*}}\left(x\right)\right]\\  & \Leftrightarrow {\mu }_{{A}_{1}^{Q*}}^{-}={\mu }_{{A}_{2}^{Q*}}^{-},{\mu }_{{A}_{1}^{Q*}}^{+}={\mu }_{{A}_{2}^{Q*}}^{+},{\nu }_{{A}_{1}^{Q*}}^{-}={\nu }_{{A}_{2}^{Q*}}^{-},{\nu }_{{A}_{1}^{Q*}}^{+}={\nu }_{{A}_{2}^{Q*}}^{+}\end{aligned}$$

4 $$\begin{aligned}{A}_{1}^{Q*}\subseteq {A}_{2}^{Q*} & \Leftrightarrow \left[{\mu }_{{A}_{1}^{Q*}}^{-},{\mu }_{{A}_{1}^{Q*}}^{+}\right]\le \left[{\mu }_{{A}_{2}^{Q*}}^{-},{\mu }_{{A}_{2}^{Q*}}^{+}\right],\left[{\nu }_{{A}_{1}^{Q*}}^{-},{\nu }_{{A}_{1}^{Q*}}^{+}\right]\ge \left[{\nu }_{{A}_{2}^{Q*}}^{-},{\nu }_{{A}_{2}^{Q*}}^{+}\right]\\ & \Leftrightarrow {\mu }_{{A}_{1}^{Q*}}^{-}\le {\mu }_{{A}_{2}^{Q*}}^{-},{\mu }_{{A}_{1}^{Q*}}^{+}\le {\mu }_{{A}_{2}^{Q*}}^{+},{\nu }_{{A}_{1}^{Q*}}^{-}\ge {\nu }_{{A}_{2}^{Q*}}^{-},{\nu }_{{A}_{1}^{Q*}}^{+}\ge {\nu }_{{A}_{2}^{Q*}}^{+}\end{aligned}$$

5 $$\begin{aligned}{A}_{1}^{Q*}\preccurlyeq {A}_{2}^{Q*} & \Leftrightarrow \left[{\mu }_{{A}_{1}^{Q*}}^{-},{\mu }_{{A}_{1}^{Q*}}^{+}\right]\preccurlyeq \left[{\mu }_{{A}_{2}^{Q*}}^{-},{\mu }_{{A}_{2}^{Q*}}^{+}\right],\left[{\nu }_{{A}_{1}^{Q*}}^{-},{\nu }_{{A}_{1}^{Q*}}^{+}\right]\succcurlyeq \left[{\nu }_{{A}_{2}^{Q*}}^{-},{\nu }_{{A}_{2}^{Q*}}^{+}\right]\\ & \Leftrightarrow {\mu }_{{A}_{1}^{Q*}}^{-}\le {\mu }_{{A}_{2}^{Q*}}^{-},{\mu }_{{A}_{1}^{Q*}}^{+}\ge {\mu }_{{A}_{2}^{Q*}}^{+},{\nu }_{{A}_{1}^{Q*}}^{-}\ge {\nu }_{{A}_{2}^{Q*}}^{-},{\nu }_{{A}_{1}^{Q*}}^{+}\le {\nu }_{{A}_{2}^{Q*}}^{+}\end{aligned}$$

### Fuzzy entropy

Fuzzy entropy is a measure used to scale the uncertainty of fuzzy elements which is come from statistics. The common fuzzy entropies are mainly as follows:

#### Definition 6

^59^ Intuitionistic fuzzy entropy: A real-valued function $E\left({A}^{I}\right):IF\left(X\right)\to \left[\mathrm{0,1}\right]$ is named as an entropy on IFS, if E satisfies all the following properties:

(I1): $$0\le E\left({A}^{I}\right)\le 1$$;
(I2): $E\left({A}^{I}\right)=0$ if ${A}^{I}$ is a crisp set;
(I3): $$E\left({A}^{I}\right)=1$$ if $${\mu }_{{A}^{I}}\left(x\right)={\nu }_{{A}^{I}}\left(x\right)={\pi }_{{A}^{I}}\left(x\right)=\frac{1}{3}$$;
(I4): $E\left({A}^{I}\right)\le E\left({B}^{I}\right)$ if $A$ is crisper than $B$, which is defined as:
  $${\mu }_{A}\left(x\right)\le {\mu }_{B}\left(x\right)\mathrm{ and }{\nu }_{A}\left(x\right)\le {\nu }_{B}\left(x\right)\mathrm{ for }max\{{\upmu }_{B},{\upnu }_{B}\}\le \frac{1}{3};$$
  $${\mu }_{A}\left(x\right)\ge {\mu }_{B}\left(x\right)\mathrm{ and }{\nu }_{A}\left(x\right)\ge {\nu }_{B}\left(x\right)\mathrm{ for }min\{{\upmu }_{B},{\upnu }_{B}\}\ge \frac{1}{3};$$

#### Definition 7

^60^ Interval-valued intuitionistic fuzzy entropy: A real-valued function $E({A}^{I*})$: $IVIF\left(X\right)\to [\mathrm{0,1}]$ is named as an entropy on IVIFS, if $E({A}^{I*})$ satisfies all the following properties:

(I^*^1): $$0\le E({A}^{I*})\le 1.$$
  (i) $E\left({A}^{I*}\right)=0$ if ${A}^{I*}$ is a crisp set.
  (ii) $$E({A}^{I*})$$ iff $${\mu }_{{A}^{I*}}\left({x}_{i}\right)={\upnu }_{{A}^{I*}}\left({x}_{i}\right)$$.

(I^*^2): $$E\left({A}^{I*}\right)=E({\left({A}^{I*}\right)}^{C}).$$
(I^*^3): $E\left({A}^{I*}\right)\le E\left({B}^{I*}\right)$ if A is crisper than B, which is defined as:
  $$ \mu_{{A^{I*} }} \left( {x_{i} } \right) \le \mu_{{B^{I*} }} \left( {x_{i} } \right), \;\upnu _{{A^{I*} }} \left( {x_{i} } \right) \ge\upnu _{{B^{I*} }} \left( {x_{i} } \right) \;{\text{for}}\;\mu_{{B^{I*} }} \left( {x_{i} } \right) \le\upnu _{{B^{I*} }} \left( {x_{i} } \right); $$
  $$ \mu_{{A^{I*} }} \left( {x_{i} } \right) \ge \mu_{{B^{I*} }} \left( {x_{i} } \right), \upnu _{{A^{I*} }} \left( {x_{i} } \right) \le\upnu _{{B^{I*} }} \left( {x_{i} } \right)\;{\text{for}}\;\mu_{{B^{I*} }} \left( {x_{i} } \right) \ge\upnu _{{B^{I*} }} \left( {x_{i} } \right); $$
  $$ \mu_{{A^{I*} }} \left( {x_{i} } \right){ \preccurlyeq }\mu_{{B^{I*} }} \left( {x_{i} } \right),{{ \nu }}_{{A^{I*} }} \left( {x_{i} } \right){ \succcurlyeq }{\upnu }_{{B^{I*} }} \left( {x_{i} } \right)\;{\text{for}}\;\mu_{{B^{I*} }} \left( {x_{i} } \right){ \preccurlyeq }{\upnu }_{{B^{I*} }} \left( {x_{i} } \right); $$
  $$ \mu_{{A^{I*} }} \left( {x_{i} } \right){ \succcurlyeq }\mu_{{B^{I*} }} \left( {x_{i} } \right),\upnu _{{A^{I*} }} \left( {x_{i} } \right){ \preccurlyeq \nu }_{{B^{I*} }} \left( {x_{i} } \right)\;{\text{for}}\;\mu_{{B^{I*} }} \left( {x_{i} } \right){ \succcurlyeq \nu }_{{B^{I*} }} \left( {x_{i} } \right). $$

### The combined compromise solution (CoCoSo) method

Yazdani et al.^58^ proposed an eclectic MADM, which is an efficient way to deal with complex decision question by using conflict analysis. CoCoSo method is implemented by the following steps for decision making after determining the alternatives and evaluation criteria:

*Step 1* The original decision matrix is normalized by Eq. (6):

6 $$ \left\{ {\begin{array}{*{20}l} {\overline{x}_{ij} = \frac{{x_{ij} - min\;x_{ij} }}{\tau }, \quad for\;benefit\;criteria;} \hfill \\ {\overline{x}_{ij} = \frac{{max\;x_{ij} - x_{ij} }}{\tau },\quad for\;cost\;criteria,} \hfill \\ \end{array} } \right. $$

where $$\tau =\mathit{max}\,{x}_{ij}-\mathit{min}\,{x}_{ij}$$.

*Step 2* Calculate the weighted sum and weighted product for each alternative based on the attribute weights by Eqs. (7–8):

7 $$ S_{\omega i} = \mathop \sum \limits_{j = 1}^{n} \omega_{j} \overline{x}_{ij} $$

8 $$ \begin{array}{*{20}c} {M_{\omega i} = \mathop \prod \limits_{j = 1}^{n} \left( {\overline{x}_{ij} } \right)^{{\omega_{j} }} } \\ \end{array} $$

*Step 3* Generate three dependent compromise scores for each alternative using the three dependent aggregation operators from different perspectives by using Eq. (9):

9 $$ \left\{ {\begin{array}{*{20}l} {S_{i}^{1} = \frac{{S_{\omega i} + M_{\omega i} }}{{\mathop \sum \nolimits_{i = 1}^{m} \left( {S_{\omega i} + M_{\omega i} } \right)}};} \hfill \\ {S_{i}^{2} = \frac{{S_{\omega i} }}{{min\,S_{\omega i} }} + \frac{{M_{\omega i} }}{{min\,M_{\omega i} }};} \hfill \\ {S_{i}^{3} = \frac{{\varepsilon S_{\omega i} + \left( {1 - \varepsilon } \right)M_{\omega i} }}{{\varepsilon max_{i} S_{\omega i} + \left( {1 - \varepsilon } \right)max_{i} M_{\omega i} }}, \quad 0 \le \varepsilon \le 1} \hfill \\ \end{array} } \right. $$

*Step 4* Calculate the three dependent compromise scores using the mixed integration operator as shown as Eq. (10):

10 $$\begin{array}{c}{S}_{i}=\frac{1}{3}\left({S}_{i}^{1}+{S}_{i}^{2}+{S}_{i}^{3}\right)+{\left({S}_{i}^{1}{S}_{i}^{2}{S}_{i}^{3}\right)}^\frac{1}{3}\end{array}$$

## A novel CoCoSo group decision making method on IVq-ROFSs

In this section, a new MAGDM method is proposed. Firstly, a new multi-source aggregation method is given for multiple IVq-ROFSs. And then a new fuzzy entropy with parameter and related weight calculation method is introduced. Finally, the new CoCoSo method on IVq-ROFS utilizing the proposed fuzzy entropy and the enhanced compromise scores is presented.

### Multi-source aggregation method on IVq-ROFSs

The information aggregation method is the first step of fuzzy MAGDM method, this paper defines a multi-source aggregation method of IVq-ROFSs inspired by Petry’s method^61^. Distinguishing from the complex aggregation operators proposed in other studies^62–64^ for the MAGDM problem, a framework for multi-source aggregation methods and two simpler approaches that satisfied the framework are presented in this section.

#### Definition 8

Let the universe $X$ be a non-empty set, then Multi-source Aggregation Interval-valued q-rung orthopair fuzzy set (MsAIVq-ROFS) is defined as follows:

11 $$ \begin{array}{*{20}c} {A_{agg}^{Q*} = \left\{ {x,\mu_{{A_{agg}^{Q*} }} \left( x \right),\nu_{{A_{agg}^{Q*} }} \left( x \right)|x \in X} \right\},} \\ \end{array} $$

where ${\mu }_{{A}_{agg}^{Q*}}=AGG\left({\mu }_{{A}_{1}^{Q*}},{\mu }_{{A}_{2}^{Q*}},\cdots ,{\mu }_{{A}_{n}^{Q*}}\right)$ and ${\nu }_{{A}_{agg}^{Q*}}=AGG\left({\nu }_{{A}_{1}^{Q*}},{\nu }_{{A}_{2}^{Q*}},\cdots ,{\nu }_{{A}_{n}^{Q*}}\right)$. $AGG\left({x}_{1},{x}_{2},\cdots ,{x}_{n}\right)$ is the aggregation function of the MD and NMD to be defined for the IVq-ROFS, expanding the function we can obtain:

$${\mu }_{{A}_{agg}^{Q*}}=AGG\left({\mu }_{{A}_{1}^{Q*}},{\mu }_{{A}_{2}^{Q*}},\cdots ,{\mu }_{{A}_{n}^{Q*}}\right)=\left[AGG\left({\mu }_{{A}_{1}^{Q*}}^{-},{\mu }_{{A}_{2}^{Q*}}^{-},\cdots ,{\mu }_{{A}_{n}^{Q*}}^{-}\right),AGG\left({\mu }_{{A}_{1}^{Q*}}^{+},{\mu }_{{A}_{2}^{Q*}}^{+},\cdots ,{\mu }_{{A}_{n}^{Q*}}^{+}\right)\right],$$

$${\nu }_{{A}_{agg}^{Q*}}=AGG\left({\nu }_{{A}_{1}^{Q*}},{\nu }_{{A}_{2}^{Q*}},\cdots ,{\nu }_{{A}_{n}^{Q*}}\right)=\left[AGG\left({\nu }_{{A}_{1}^{Q*}}^{-},{\nu }_{{A}_{2}^{Q*}}^{-},\cdots ,{\nu }_{{A}_{n}^{Q*}}^{-}\right),AGG\left({\nu }_{{A}_{1}^{Q*}}^{+},{\nu }_{{A}_{2}^{Q*}}^{+},\cdots ,{\nu }_{{A}_{n}^{Q*}}^{+}\right)\right] ,$$

and ${\mu }_{{A}_{n}^{Q*}}^{+}$,${\mu }_{{A}_{n}^{Q*}}^{-}$,${\nu }_{{A}_{n}^{Q*}}^{+}$,${\nu }_{{A}_{n}^{Q*}}^{-}$ are the upper and lower bounds of the MD and NMD of IVq-ROFS. Next, considering the different requirements needed by different decision makers in a complex decision environment, this paper proposes two aggregation functions for IVq-ROFSs.

#### Definition 9

For n IVq-ROFSs of the same universe $X$, the compromised MsAIVq-ROFS is as follows:

12 $$\begin{array}{c}{A}_{pos}^{Q*}=\left\{\langle x,{\mu }_{{A}_{pos}^{Q*}}\left(x\right),{\nu }_{{A}_{pos}^{Q*}}\left(x\right)\rangle |x\in X\right\}\end{array}$$

13 $$\begin{array}{c}{\mu }_{{A}_{pos}^{Q*}}=\left[\frac{\sum_{i=1}^{n}{\mu }_{{A}_{i}^{Q*}}^{-}}{n},\frac{\sum_{i=1}^{n}{\mu }_{{A}_{i}^{Q*}}^{+}}{n}\right],{\nu }_{{A}_{pos}^{Q*}}=\left[\frac{\sum_{i=1}^{n}{\nu }_{{A}_{i}^{Q*}}^{-}}{n},\frac{\sum_{i=1}^{n}{\nu }_{{A}_{i}^{Q*}}^{+}}{n}\right]\end{array}$$

${S}_{{\mu }_{{A}_{i}^{Q*}}^{+}}$, ${S}_{{\mu }_{{A}_{i}^{Q*}}^{-}}$, ${S}_{{\nu }_{{A}_{i}^{Q*}}^{+}}$, ${S}_{{\nu }_{{A}_{i}^{Q*}}^{-}}$ are the sets of upper and lower bounds of the MD and NMD to be aggregated. ${\mu }_{{A}_{i}^{Q*}}^{+}\in {S}_{{\mu }_{{A}_{i}^{Q*}}^{+}}$, ${\mu }_{{A}_{i}^{Q*}}^{-}\in {S}_{{\mu }_{{A}_{i}^{Q*}}^{-}}$, ${\nu }_{{A}_{i}^{Q*}}^{+}\in {S}_{{\nu }_{{A}_{i}^{Q*}}^{+}}$, ${\nu }_{{A}_{i}^{Q*}}^{-}\in {S}_{{\nu }_{{A}_{i}^{Q*}}^{-}}$.

#### Definition 10

For n IVq-ROFSs of the same object set X, the inclusive MsAIVq-ROFS is as follows:

14 $$\begin{array}{c}{A}_{neg}^{Q*}=\left\{\langle x,{\mu }_{{A}_{neg}^{Q*}}\left(x\right),{\nu }_{{A}_{neg}^{Q*}}\left(x\right)\rangle |x\in X\right\}\end{array}$$

15 $$\begin{array}{c}{\mu }_{{A}_{neg}^{Q*}}=\left[min\left({\mu }_{{A}_{s}^{Q*}}^{-}\right),max\left({\mu }_{{A}_{s}^{Q*}}^{+}\right)\right],{\nu }_{{A}_{neg}^{Q*}}=\left[min\left({\nu }_{{A}_{s}^{Q*}}^{-}\right),max\left({\nu }_{{A}_{s}^{Q*}}^{+}\right)\right].\end{array}$$

These two approaches to aggregation of information sources are illustrated below by a supplier selection problem.

#### Example 1

Let the existing supplier object set be $O=\left\{{O}_{1},{O}_{2},{O}_{3},{O}_{4},{O}_{5},{O}_{6}\right\}$, data information sources $S=\left\{{S}_{1},{S}_{2}\right\}$, attribute set $A=\left\{{a}_{1},{a}_{2},{a}_{3},{a}_{4},{a}_{5}\right\}$. Here ${a}_{1}$ is delivery capability, ${a}_{2}$ is product price, ${a}_{3}$ is technical support, ${a}_{4}$ is service level, ${a}_{5}$ is inventory level. The original IVq-ROFSs given by the two information sources are Tables 1 and 2 respectively.

**Table 1:** The original data set given by ${S}_{1}$.

${{{S}}}_{1}$	${{{a}}}_{1}$	${{{a}}}_{2}$	${{{a}}}_{3}$	${{{a}}}_{4}$	${{{a}}}_{5}$
${O}_{1}$	<[0.1, 0.3], [0.4, 0.5]>	<[0.4, 0.6], [0.2, 0.5]>	<[0.2, 0.4], [0.4, 0.6]>	<[0.2, 0.4], [0.2, 0.6]>	<[0.1, 0.3], [0.1, 0.3]>
${O}_{2}$	<[0.5, 0.8], [0.1, 0.3]>	<[0.3, 0.5], [0.3, 0.8]>	<[0.4, 0.6], [0.2, 0.4]>	<[0.4, 0.6], [0.2, 0.5]>	<[0.1, 0.3], [0.1, 0.3]>
${O}_{3}$	<[0.8, 0.9], [0.3, 0.4]>	<[0.4, 0.6], [0.2, 0.6]>	<[0.1, 0.2], [0.6, 0.8]>	<[0.3, 0.5], [0.1, 0.2]>	<[0.1, 0.3], [0.1, 0.3]>
${O}_{4}$	<[0.5, 0.6], [0.2, 0.4]>	<[0.4, 0.6], [0.4, 0.5]>	<[0.2, 0.6], [0.4, 0.8]>	<[0.4, 0.6], [0.2, 0.5]>	<[0.3, 0.4], [0.5, 0.7]>
${O}_{5}$	<[0.6, 0.7], [0.3, 0.5]>	<[0.1, 0.3], [0.3, 0.6]>	<[0.4, 0.8], [0.1, 0.4]>	<[0.1, 0.3], [0.1, 0.3]>	<[0.3, 0.6], [0.2, 0.7]>
${O}_{6}$	<[0.4, 0.7], [0.3, 0.5]>	<[0.4, 0.7], [0.2, 0.4]>	<[0.2, 0.4], [0.5, 0.8]>	<[0.1, 0.3], [0.1, 0.3]>	<[0.3, 0.8], [0.2, 0.5]>

**Table 2:** The original data set given by ${S}_{2}$.

	${{{a}}}_{1}$	${{{a}}}_{2}$	${{{a}}}_{3}$	${{{a}}}_{4}$	${{{a}}}_{5}$
${O}_{1}$	<[0.2, 0.4], [0.2, 0.6]>	<[0.4, 0.7], [0.2, 0.3]>	<[0.3, 0.5], [0.2, 0.3]>	<[0.3, 0.4], [0.3, 0.5]>	<[0.2, 0.4], [0.2, 0.3]>
${O}_{2}$	<[0.3, 0.5], [0.2, 0.3]>	<[0.4, 0.5], [0.2, 0.6]>	<[0.1, 0.2], [0.8, 0.9]>	<[0.1, 0.2], [0.3, 0.4]>	<[0.5, 0.7], [0.1, 0.3]>
${O}_{3}$	<[0.5, 0.6], [0.2, 0.3]>	<[0.2, 0.4], [0.5, 0.6]>	<[0.3, 0.7], [0.2, 0.8]>	<[0.6, 0.7], [0.1, 0.3]>	<[0.3, 0.6], [0.1, 0.3]>
${O}_{4}$	<[0.3, 0.5], [0.1, 0.2]>	<[0.2, 0.6], [0.3, 0.4]>	<[0.1, 0.2], [0.3, 0.5]>	<[0.2, 0.3], [0.1, 0.6]>	<[0.4, 0.6], [0.2, 0.3]>
${O}_{5}$	<[0.2, 0.8], [0.1, 0.2]>	<[0.5, 0.7], [0.2, 0.6]>	<[0.2, 0.6], [0.3, 0.6]>	<[0.2, 0.4], [0.1, 0.4]>	<[0.1, 0.2], [0.7, 0.8]>
${O}_{6}$	<[0.1, 0.3], [0.3, 0.5]>	<[0.6, 0.8], [0.2, 0.4]>	<[0.4, 0.6], [0.2, 0.3]>	<[0.2, 0.6], [0.2, 0.4]>	<[0.2, 0.3], [0.1, 0.3]>

The compromised MsAIVq-ROFS and inclusive MsAIVq-ROFS on the Tables 1 and 2 are obtained using the formulas of Definition 9 and Definition 10 as Tables 3 and 4 shown.

**Table 3:** Compromised MsAIVq-ROFS.

	${{{a}}}_{1}$	${{{a}}}_{2}$	${{{a}}}_{3}$	${{{a}}}_{4}$	${{{a}}}_{5}$
${S}_{1}$	<[0.15, 0.35], [0.3, 0.55]>	<[0.4, 0.65], [0.2, 0.4]>	<[0.25, 0.45], [0.3, 0.45]>	<[0.25, 0.4], [0.25, 0.55]>	<[0.15, 0.35], [0.15, 0.3]>
${S}_{2}$	<[0.4, 0.65], [0.15, 0.3]>	<[0.35, 0.5], [0.25, 0.7]>	<[0.25, 0.4], [0.5, 0.65]>	<[0.25, 0.4], [0.25, 0.45]>	<[0.3, 0.5], [0.1, 0.3]>
${S}_{3}$	<[0.65, 0.75], [0.25, 0.35]>	<[0.3, 0.5], [0.35, 0.6]>	<[0.2, 0.45], [0.4, 0.8]>	<[0.45, 0.6], [0.1, 0.25]>	<[0.2, 0.45], [0.1, 0.3]>
${S}_{4}$	<[0.4, 0.55], [0.15, 0.3]>	<[0.3, 0.6], [0.35, 0.45]>	<[0.15, 0.4], [0.35, 0.65]>	<[0.3, 0.45], [0.15, 0.55]>	<[0.35, 0.5], [0.35, 0.5]>
${S}_{5}$	<[0.4, 0.75], [0.2, 0.35]>	<[0.3, 0.5], [0.25, 0.6]>	<[0.3, 0.7], [0.2, 0.5]>	<[0.15, 0.35], [0.1, 0.35]>	<[0.2, 0.4], [0.45, 0.75]>
${S}_{6}$	<[0.1, 0.3], [0.3, 0.5]>	<[0.6, 0.8], [0.2, 0.4]>	<[0.4, 0.6], [0.2, 0.3]>	<[0.2, 0.6], [0.2, 0.4]>	<[0.2, 0.3], [0.1, 0.3]>

**Table 4:** Inclusive MsAIVq-ROFS.

	${{{a}}}_{1}$	${{{a}}}_{2}$	${{{a}}}_{3}$	${{{a}}}_{4}$	${{{a}}}_{5}$
${S}_{1}$	<[0.1, 0.4], [0.2, 0.6]>	<[0.4, 0.7], [0.2, 0.5]>	<[0.2, 0.5], [0.2, 0.6]>	<[0.2, 0.4], [0.2, 0.6]>	<[0.1, 0.4], [0.1, 0.3]>
${S}_{2}$	<[0.3, 0.8], [0.1, 0.3]>	<[0.3, 0.5], [0.2, 0.8]>	<[0.1, 0.6], [0.2, 0.9]>	<[0.1, 0.6], [0.2, 0.5]>	<[0.1, 0.7], [0.1, 0.3]>
${S}_{3}$	<[0.5, 0.9], [0.2, 0.4]>	<[0.2, 0.6], [0.2, 0.6]>	<[0.1, 0.7], [0.2, 0.8]>	<[0.3, 0.7], [0.1, 0.3]>	<[0.1, 0.6], [0.1, 0.3]>
${S}_{4}$	<[0.3, 0.6], [0.1, 0.4]>	<[0.2, 0.6], [0.3, 0.5]>	<[0.1, 0.6], [0.3, 0.8]>	<[0.2, 0.6], [0.1, 0.6]>	<[0.3, 0.6], [0.2, 0.7]>
${S}_{5}$	<[0.2, 0.8], [0.1, 0.5]>	<[0.1, 0.7], [0.2, 0.6]>	<[0.2, 0.8], [0.1, 0.6]>	<[0.1, 0.4], [0.1, 0.4]>	<[0.1, 0.6], [0.2, 0.8]>
${S}_{6}$	<[0.1, 0.7], [0.3, 0.5]>	<[0.4, 0.8], [0.2, 0.4]>	<[0.2, 0.6], [0.2, 0.8]>	<[0.1, 0.6], [0.1, 0.4]>	<[0.2, 0.8], [0.1, 0.5]>

Among them, inclusive MsAIVq-ROFS may have a certain problem that the sum of the squares of the upper limit of MD and the upper limit of NMD of the elements to be aggregated may be greater than 1. Therefore, we need to restrict the merge aggregation by setting the upper limit of NMD degree to $\left\lfloor {\sqrt[q]{{1 - \left( {\mu_{{A^{{Q^{*} }} }}^{ - } } \right)^{q} }}} \right\rfloor$ when the sum of the squares of the upper limit of MD and the upper limit of NMD of the aggregated elements is greater than 1, so as to solve this problem.

From the comparison of information before and after aggregation, it can be seen that the aggregated MsAIVq-ROFS has the characteristics of two data information sources at the same time, and two methods bring radical and conservative strategies to the subsequent decision-making ideas. And compared with complex aggregation operators in recent researches^55^, these two methods are more concise and more accessible to calculate.

In summary, the two aggregation methods for IVq-ROFSs proposed in this paper perform different operations on the upper and lower values of the interval values, respectively, and obtain two types of IVq-ROFSs decision datasets that can provide different decision-making opinions. The compromised method calculates the mean value of the upper and lower values of multiple IVq-ROFSs to reduce the range of intervals that can be calculated and contain the original data set’s characteristic information to achieve a more decision-making idea. The inclusive method calculates the maximum value of the upper and lower values of multiple IVq-ROFSs to ensure that all the original data set information can be includedto achieve a more accurate decision-making idea. Both approaches will work for different scenarios.

### A new fuzzy entropy with parameter and related weight calculation method on IVq-ROFS

Objective attribute weight calculation is the second step of fuzzy MAGDM, given the “Fuzzy entropy” section conditions, a new fuzzy entropy with parameters for IVq-ROFS is proposed here:

#### Definition 11

Interval-valued q-rung orthopair fuzzy entropy: A real-valued function E: $IVq-ROF\left(X\right)\to [\mathrm{0,1}]$ is named as an entropy on IVq-ROFS, if E satisfies all the following properties:

(Q1): $$0\le E({A}^{Q*})\le 1$$.
  (i) $E\left({A}^{Q*}\right)=0$ if ${A}^{Q*}$ is a crisp set.
  (ii) $$E\left({A}^{Q*}\right)=1$$ iff $${\mu }_{{A}^{Q*}}\left({x}_{i}\right)={\upnu }_{{A}^{Q*}}\left({x}_{i}\right)=[\sqrt[q]{k},\sqrt[q]{k}]$$.
(Q2): $$E\left({A}^{Q*}\right)=E({\left({A}^{Q*}\right)}^{C})$$.
(Q3): $E\left({A}^{Q*}\right)\le E\left({B}^{Q*}\right)$ if ${A}^{Q*}$ is crisper than ${B}^{Q*}$,which is defined as:
  $${\mu }_{{B}^{Q*}}\left({x}_{i}\right)\ge {\mu }_{{A}^{Q*}}\left({x}_{i}\right)\ge [\sqrt[q]{k},\sqrt[q]{k}]\quad\text{and}\quad{\upnu }_{{B}^{Q*}}\left({x}_{i}\right)\ge {\upnu }_{{A}^{Q*}}\left({x}_{i}\right)\ge [\sqrt[q]{k},\sqrt[q]{k}]$$
  where $k\in [\mathrm{0,1}]$. The parameter k is a balance parameter to improve the flexibility of the fuzzy entropy.

#### Definition 12

Let the theoretical domain X be a nonempty set X and ${A}^{Q*}=\{<{x}_{i},{\mu }_{{A}^{Q*}}\left({x}_{i}\right),{\nu }_{{A}^{Q*}}\left({x}_{i}\right)>|{x}_{i}\in X\}$ be an IVq-ROFS, the new entropy formula of IVq-ROFS is as follow:

16 $$\begin{array}{c}E\left({A}^{Q*}\right)=\frac{1}{n}\sum_{i=1}^{n}\frac{\left[1-{\mu }_{{A}^{Q*}}^{\searrow }-{\upnu }_{{A}^{Q*}}^{\searrow }\right]+\left[1-{\mu }_{{A}^{Q*}}^{\nearrow }-{\upnu }_{{A}^{Q*}}^{\nearrow }\right]}{2},\end{array}$$

where $${\mu }_{{A}^{Q*}}^{\searrow }={\text{min}}\left(\left|{{\mu }_{{A}^{Q*}}^{-}}^{q}\left({{\text{x}}}_{i}\right)-{\text{k}}\right|,\left|{{\mu }_{{A}^{Q*}}^{+}}^{q}\left({{\text{x}}}_{i}\right)-{\text{k}}\right|\right),$$ $${\upnu }_{{A}^{Q*}}^{\searrow }={\text{min}}\left(\left|{{\upnu }_{{A}^{Q*}}^{-}}^{q}\left({{\text{x}}}_{i}\right)-{\text{k}}\right|,\left|{{\upnu }_{{A}^{Q*}}^{+}}^{q}\left({{\text{x}}}_{i}\right)-{\text{k}}\right|\right),$$ $${\mu }_{{A}^{Q*}}^{\nearrow }={\text{max}}\left(\left|{{\mu }_{{A}^{Q*}}^{-}}^{q}\left({{\text{x}}}_{i}\right)-{\text{k}}\right|,\left|{{\mu }_{{A}^{Q*}}^{+}}^{q}\left({{\text{x}}}_{i}\right)-{\text{k}}\right|\right),$$ $${\upnu }_{{A}^{Q*}}^{\nearrow }={\text{max}}(\left|{{\upnu }_{{A}^{Q*}}^{-}}^{q}\left({{\text{x}}}_{i}\right)-{\text{k}}\right|,|{{\upnu }_{{A}^{Q*}}^{+}}^{q}\left({{\text{x}}}_{i}\right)-{\text{k}}|)$$.

#### Proof

(Q1)(i): If ${A}^{Q*}$ is a crisp set, then we have ${\mu }_{{A}^{Q*}}=[\mathrm{0,0}]$ and ${\upnu }_{{A}^{Q*}}=[\mathrm{1,1}]$ or ${\mu }_{{A}^{Q*}}=[\mathrm{1,1}]$ and ${\upnu }_{{A}^{Q*}}=[\mathrm{0,0}]$
  When ${\mu }_{{A}^{Q*}}=[\mathrm{0,0}]$ and ${\upnu }_{{A}^{Q*}}=[\mathrm{1,1}]$, $E\left({A}^{Q*}\right)=\frac{1}{n}\sum_{i=1}^{n}\frac{\left[1-k-(1-k)\right]+\left[1-k-(1-k)\right]}{2}=0.$ When ${\mu }_{{A}^{Q*}}=[\mathrm{1,1}]$ and ${\upnu }_{{A}^{Q*}}=[\mathrm{0,0}]$,$E\left({A}^{Q*}\right)=\frac{1}{n}\sum_{i=1}^{n}\frac{\left[1-k-(1-k)\right]+\left[1-k-(1-k)\right]}{2}=0.$
(Q1)(ii): Firstly, sufficiency is proofed as follows: when $E\left({A}^{Q*}\right)=\frac{1}{n}\sum_{i=1}^{n}\frac{\left[1-{\mu }_{{A}^{Q*}}^{\searrow }-{\upnu }_{{A}^{Q*}}^{\searrow }\right]+\left[1-{\mu }_{{A}^{Q*}}^{\nearrow }-{\upnu }_{{A}^{Q*}}^{\nearrow }\right]}{2}=1$, so we have ${\mu }_{{A}^{Q*}}^{\searrow }={\upnu }_{{A}^{Q*}}^{\searrow }={\mu }_{{A}^{Q*}}^{\nearrow }={\upnu }_{{A}^{Q*}}^{\nearrow }=0$. It means that, ${\text{min}}\left(\left|{{\mu }_{{A}^{Q*}}^{-}}^{q}\left({{\text{x}}}_{i}\right)-{\text{k}}\right|,\left|{{\mu }_{{A}^{Q*}}^{+}}^{q}\left({{\text{x}}}_{i}\right)-{\text{k}}\right|\right)$ $={\text{min}}\left(\left|{{\upnu }_{{A}^{Q*}}^{-}}^{q}\left({{\text{x}}}_{i}\right)-{\text{k}}\right|,\left|{{\upnu }_{{A}^{Q*}}^{+}}^{q}\left({{\text{x}}}_{i}\right)-{\text{k}}\right|\right)$ $={\text{max}}\left(\left|{{\mu }_{{A}^{Q*}}^{-}}^{q}\left({{\text{x}}}_{i}\right)-{\text{k}}\right|,\left|{{\mu }_{{A}^{Q*}}^{+}}^{q}\left({{\text{x}}}_{i}\right)-{\text{k}}\right|\right)$ $={\text{max}}\left(\left|{{\upnu }_{{A}^{Q*}}^{-}}^{q}\left({{\text{x}}}_{i}\right)-{\text{k}}\right|,\left|{{\upnu }_{{A}^{Q*}}^{+}}^{q}\left({{\text{x}}}_{i}\right)-{\text{k}}\right|\right)=0.$ So we have ${\mu }_{{A}^{Q*}}\left({x}_{i}\right)={\upnu }_{{A}^{Q*}}\left({x}_{i}\right)=[\sqrt[q]{k},\sqrt[q]{k}]$.Secondly, necessity is proofed as follows: when ${\mu }_{{A}^{Q*}}\left({x}_{i}\right)={\upnu }_{{A}^{Q*}}\left({x}_{i}\right)=[\sqrt[q]{k},\sqrt[q]{k}]$, then we have ${\mu }_{{A}^{Q*}}^{\searrow }={\upnu }_{{A}^{Q*}}^{\searrow }={\mu }_{{A}^{Q*}}^{\nearrow }={\upnu }_{{A}^{Q*}}^{\nearrow }=0$. So we have $E\left({A}^{Q*}\right)=\frac{1}{n}\sum_{i=1}^{n}\frac{\left[1-{\mu }_{{A}^{Q*}}^{\searrow }-{\upnu }_{{A}^{Q*}}^{\searrow }\right]+\left[1-{\mu }_{{A}^{Q*}}^{\nearrow }-{\upnu }_{{A}^{Q*}}^{\nearrow }\right]}{2}=1.$
(Q2): $$E\left({A}^{Q*}\right)=\frac{1}{n}\sum_{i=1}^{n}\frac{\left[1-{\mu }_{{A}^{Q*}}^{\searrow }-{\upnu }_{{A}^{Q*}}^{\searrow }\right]+\left[1-{\mu }_{{A}^{Q*}}^{\nearrow }-{\upnu }_{{A}^{Q*}}^{\nearrow }\right]}{2}=\frac{1}{n}\sum_{i=1}^{n}\frac{\left[1-{\upnu }_{{A}^{Q*}}^{\searrow }-{\mu }_{{A}^{Q*}}^{\searrow }\right]+\left[1-{\upnu }_{{A}^{Q*}}^{\nearrow }-{\mu }_{{A}^{Q*}}^{\nearrow }\right]}{2}=E({\left({A}^{Q*}\right)}^{C})$$
(Q3): $E\left({A}^{Q*}\right)-E\left({B}^{Q*}\right)=$ $\frac{1}{n}\sum_{i=1}^{n}\frac{\left[1-{\mu }_{{A}^{Q*}}^{\searrow }-{\upnu }_{{A}^{Q*}}^{\searrow }\right]+\left[1-{\mu }_{{A}^{Q*}}^{\nearrow }-{\upnu }_{{A}^{Q*}}^{\nearrow }\right]-\left[1-{\mu }_{{B}^{Q*}}^{\searrow }-{\upnu }_{{B}^{Q*}}^{\searrow }\right]+\left[1-{\mu }_{{B}^{Q*}}^{\nearrow }-{\upnu }_{{B}^{Q*}}^{\nearrow }\right]}{2}=$ $\frac{1}{n}\sum_{i=1}^{n}\frac{\left[{(\mu }_{{B}^{Q*}}^{\searrow }-{\mu }_{{A}^{Q*}}^{\searrow })+({\upnu }_{{B}^{Q*}}^{\searrow }-{\upnu }_{{A}^{Q*}}^{\searrow })\right]+\left[\left({\mu }_{{B}^{Q*}}^{\nearrow }-{\mu }_{{A}^{Q*}}^{\nearrow }\right)+({\upnu }_{{B}^{Q*}}^{\nearrow }-{\upnu }_{{A}^{Q*}}^{\nearrow })\right]}{2}$. According to this equation, we know that the value of $E\left({A}^{Q*}\right)-E\left({B}^{Q*}\right)$ depends on $\left[{(\mu }_{{B}^{Q*}}^{\searrow }-{\mu }_{{A}^{Q*}}^{\searrow })+({\upnu }_{{B}^{Q*}}^{\searrow }-{\upnu }_{{A}^{Q*}}^{\searrow })\right]+\left[\left({\mu }_{{B}^{Q*}}^{\nearrow }-{\mu }_{{A}^{Q*}}^{\nearrow }\right)+({\upnu }_{{B}^{Q*}}^{\nearrow }-{\upnu }_{{A}^{Q*}}^{\nearrow })\right]$. When ${\mu }_{{B}^{Q*}}\left({x}_{i}\right)\ge {\mu }_{{A}^{Q*}}\left({x}_{i}\right)\ge [\sqrt[q]{k},\sqrt[q]{k}]$ and ${\upnu }_{{B}^{Q*}}\left({x}_{i}\right)\ge {\upnu }_{{A}^{Q*}}\left({x}_{i}\right)\ge [\sqrt[q]{k},\sqrt[q]{k}]$, then $\left[{(\mu }_{{B}^{Q*}}^{\searrow }-{\mu }_{{A}^{Q*}}^{\searrow })+({\upnu }_{{B}^{Q*}}^{\searrow }-{\upnu }_{{A}^{Q*}}^{\searrow })\right]+\left[\left({\mu }_{{B}^{Q*}}^{\nearrow }-{\mu }_{{A}^{Q*}}^{\nearrow }\right)+({\upnu }_{{B}^{Q*}}^{\nearrow }-{\upnu }_{{A}^{Q*}}^{\nearrow })\right]\ge 0$. It means that $E\left({A}^{Q*}\right)-E\left({B}^{Q*}\right)\ge 0$*.*

It can be seen that the proposed new fuzzy entropy satisfies the condition that can represent the fuzziness of any IVq-ROFSs.

The traditional entropy weight calculation method generally uses Shannon entropy to calculate entropy values, a quantitative measure of information. However, fuzzy entropy is used for weight calculation instead of Shannon entropy to fit the fuzzy idea. Fuzzy entropy is based on fuzzy theory and expresses the quantitative fuzzy degree of a certain fuzzy element. Based on the fuzzy entropy formula proposed above and the traditional entropy weight method, an IVq-ROFS-based attribute weight calculation method is proposed.

IVq-ROFS-based attribute weight calculation method is introduced as follow:

Calculation of the corresponding attribute based on Eq. (16) is given by:

17 $$\begin{array}{c}{\omega }_{j}=\frac{1-{E}_{j}^{({A}^{Q*})}}{n-\sum_{i=1}^{n}{E}_{i}^{({A}^{Q*})}}\end{array}$$

Further normalization is performed to obtain the final normalized attribute weights:

18 $$\begin{array}{c}{\overline{\omega }}_{j}=\frac{{\omega }_{j}}{\sum_{i=1}^{j}{\omega }_{i}}\end{array}$$

### A new CoCoSo method based on IVq-ROFS

The core of the fuzzy MAGDM method is the ranking calculation of alternatives, and the CoCoSo method is optimized by the multi-source aggregation method and IVq-ROFS-based attribute weight calculation method proposed in “Multi-source Aggregation method on IVq-ROFSs” and “A new fuzzy entropy with parameter and related weight calculation method on IVq-ROFS” sections. The original CoCoSo method has some defects. We find that because there is no normalization in the calculation of the eclectic score ${S}_{i}^{2}$, so that ${S}_{i}^{2}\ge 2$, at the same time for the same set of data $0\le {S}_{i}^{1}\le 1$, $0\le {S}_{i}^{3}\le 1$, so in the final the ranking of alternatives will lead to the ranking results dominated by ${S}_{i}^{2}$. To apply the new CoCoSo method to the IVq-ROFS environment, we calculate the fuzzy entropy value of each fuzzy element and use the entropy value as the basic element of the decision matrix for subsequent decisions. Based on the IVq-ROFS-fuzzy entropy weight method and the improvement of the hybrid score operator of three dependent such scores, we propose a new CoCoSo method in this section.

*Step 1* Standardization. The decision matrix is constructed using the new fuzzy entropy Eq. (16) for each attribute, while making $k=\frac{1}{3}$ in Eq. (16), using normalized fuzzy entropy values for benefit attributes and normalized fuzzy entropy complements for cost attributes, the standardization process is as follows.

19 $$ \left\{ {\begin{array}{*{20}l} {r_{ij} = E^{{k = \frac{1}{3}}} \left( {A^{Q*} } \right),\quad for \;benefit\;criteria;} \hfill \\ {r_{ij} = 1 - E^{{k = \frac{1}{3}}} \left( {A^{Q*} } \right),\quad for\;cost\;criteria,} \hfill \\ \end{array} } \right. $$

20 $$\begin{array}{c}{\overline{r} }_{ij}=\frac{{r}_{ij}}{\sum_{i=1}^{n}{r}_{ij}}\end{array}$$

*Step 2* Calculate the attribute weights. Use the Eq. (16) to calculate the weight of the sum of the original attribute fuzzy values for each attribute. The attribute fuzzy entropy is normalized by Eq. (21).

21 $$\begin{array}{c}{E}_{j}^{\left({A}^{Q*}\right)}=\frac{{E}_{j}^{\left({A}^{Q*}\right)}}{\sum_{i=1}^{n}{E}_{j}^{\left({A}^{Q*}\right)}}\end{array}$$

From the above equation, the normalized fuzzy entropy of each attribute is obtained, and the attribute weights are calculated using Eqs. (22, 18).

22 $$\begin{array}{c}{\omega }_{j}=\frac{1-{\overline{E} }_{j}^{\left({A}^{Q*}\right)}}{n-\sum_{i=1}^{n}{\overline{E} }_{i}^{\left({A}^{Q*}\right)}}\end{array}$$

*Step 3* Calculate the arithmetic weighted sum and arithmetic weighted product as shown as Eqs. (23, 24) for each alternative.

23 $$\begin{array}{c}{\overline{S} }_{\omega i}=\sum_{j=1}^{n}{\omega }_{i}{\overline{E} }_{j}^{\left({A}^{Q*}\right)}\end{array}$$

24 $$\begin{array}{c}{\overline{M} }_{\omega i}=\prod_{j=1}^{n}{\left({\overline{E} }_{j}^{\left({A}^{Q*}\right)}\right)}^{{\omega }_{i}}\end{array}$$

*Step 4* Generate three dependent compromise scores as shown as Eq. (25) for each alternative.

25 $$ \left\{ {\begin{array}{*{20}l} {\overline{S}_{i}^{1} = \frac{{\overline{S}_{\omega i} + \overline{M}_{\omega i} }}{{\mathop \sum \nolimits_{i = 1}^{m} \left( {\overline{S}_{\omega i} + \overline{M}_{\omega i} } \right)}};} \hfill \\ {\overline{S}_{i}^{2} = \frac{{\overline{S}_{\omega i} }}{{min \overline{S}_{\omega i} }} + \frac{{\overline{M}_{\omega i} }}{{min \overline{M}_{\omega i} }};} \hfill \\ {\overline{S}_{i}^{3} = \frac{{\varepsilon \overline{S}_{\omega i} + \left( {1 - \varepsilon } \right)\overline{M}_{\omega i} }}{{\varepsilon max_{i} \overline{S}_{\omega i} + \left( {1 - \varepsilon } \right)max_{i} \overline{M}_{\omega i} }},\quad 0 \le \varepsilon \le 1} \hfill \\ \end{array} } \right. $$

where $\varepsilon$ is an equilibrium parameter in the compromise scores to enhance the universality of the proposed method.

*Step 5* Based on the above three dependent compromise scores, the final score of each alternative is calculated using the improved compromise score function as shown as Eq. (26).

26 $$\begin{array}{c}{S}_{i}=\frac{1}{3}\left({S}_{i}^{1}+{S}_{i}^{2}+{S}_{i}^{3}\right)+{\left({S}_{i}^{1}{S}_{i}^{2}{S}_{i}^{3}\right)}^\frac{1}{3}\end{array}$$

**Figure 2:**
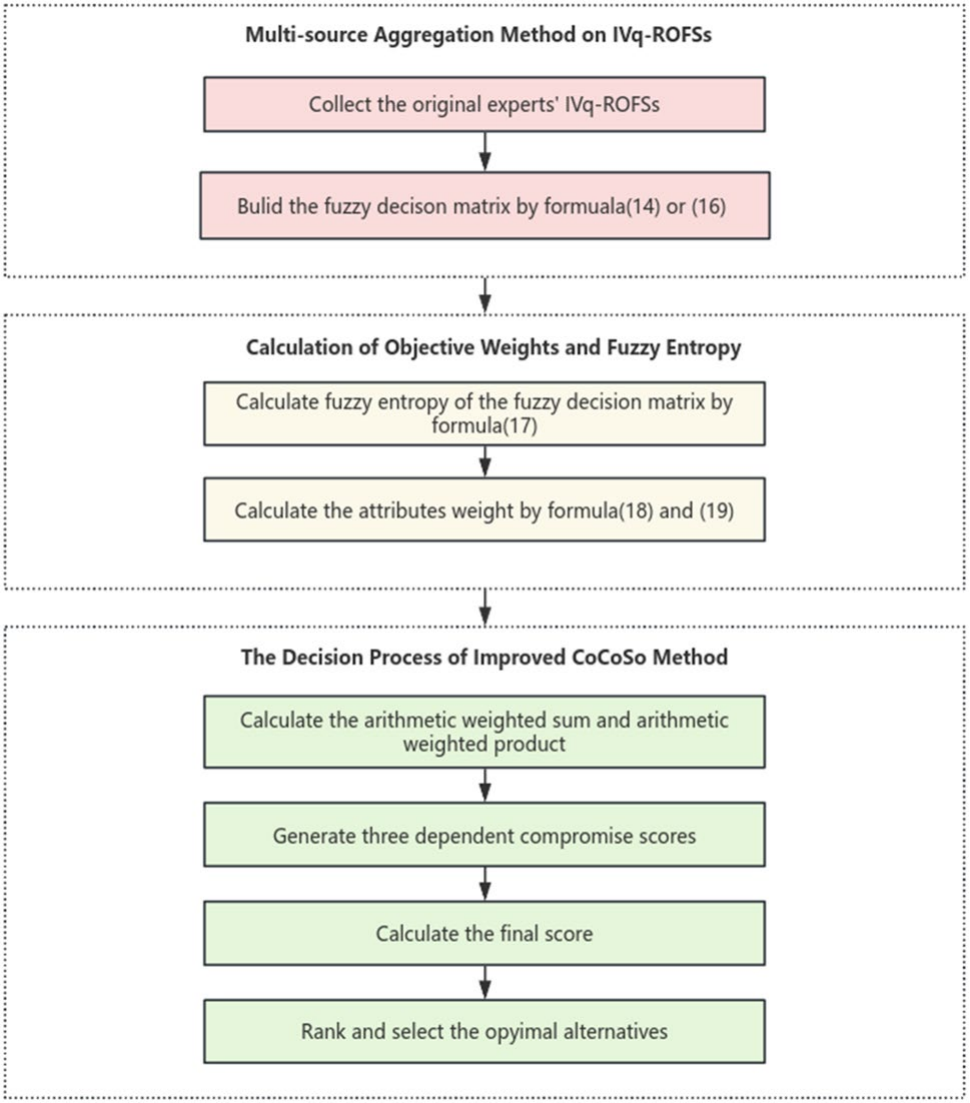
The process of proposed method.

The results of the alternative ranking are in descending order according to the final performance index, which can get more accurate ranking results. The process of proposed method is shown as Fig. 2.

## Case Study: Sepsis diagnosis

In this section, the applicability of the proposed improved CoCoSo method will be demonstrated in terms of Sepsis diagnosis.

### The description of Sepsis diagnosis

Sepsis^65,66^ is a vicious condition with a high morbidity and mortality rate. Early recognition and treatment of sepsis is the key to reducing sepsis mortality, which has been high in morbidity and mortality over the past decades. Early and accurate morbidity prediction can lead to more aggressive and effective treatment of sepsis, thereby reducing the incidence of multiorgan dysfunction and significantly reducing mortality. Early recognition can be complex due to the complexity of the disease in the clinical setting. Typically, sepsis can be judged based on attributes^67–71^ such as temperature, pulse, pain level, SaO2, blood pressure, level of consciousness, capillary refill time, urine output, and degree of ScvO2/base deficiency, as shown in Table 5. The pain level and level of consciousness are subjective attributes, for initial diagnosis in case of suspected infection. To ensure data integrity while considering expert ability, we will use sepsis feature datasets from experts P_1_ and P_2_.

**Table 5:** Description of attributes.

Attributes	Description
${C}_{1}$	Temperature
${C}_{2}$	Pulse
${C}_{3}$	Pain level
${C}_{4}$	SaO2
${C}_{5}$	Blood pressure
${C}_{6}$	Level of consciousness
${C}_{7}$	Capillary refill time
${C}_{8}$	Urine output
${C}_{9}$	Degree of ScvO2/base deficiency

### The decision process of Sepsis diagnosis

The effectiveness of the method proposed in this paper for early sepsis diagnosis is demonstrated below. Simulation dataset is used for the research data in this paper. These datasets are shown in Tables 6 and 7.

**Table 6:** Sepsis characteristics on IVq-ROFS X_1_ by Expert P_1_.

	${{{A}}}_{1}$	${{{A}}}_{2}$	${{{A}}}_{3}$	${{{A}}}_{4}$	${{{A}}}_{5}$	${{{A}}}_{6}$
${C}_{1}$	<(0.2, 0.4), (0.5, 0.6)>	<(0.1, 0.2), (0.6, 0.7)>	<(0.1, 0.2), (0.6, 0.8)>	<(0.4, 0.6), (0.2, 0.4)>	<(0.2, 0.6), (0.7, 0.8)>	<(0.1, 0.4), (0.7, 0.8)>
${C}_{2}$	<(0.2, 0.3), (0.4, 0.5)>	<(0.1, 0.3), (0.2, 0.6)>	<(0.1, 0.2), (0.1, 0.7)>	<(0.1, 0.2), (0.4, 0.5)>	<(0.4, 0.8), (0.1, 0.2)>	<(0.1, 0.4), (0.7, 0.8)>
${C}_{3}$	<(0.3, 0.4), (0.5, 0.8)>	<(0.2, 0.5), (0.5, 0.6)>	<(0.3, 0.6), (0.6, 0.7)>	<(0.2, 0.3), (0.5, 0.6)>	<(0.1.0.5), (0.4, 0.7)>	<(0.3, 0.4), (0.65, 0.7)>
${C}_{4}$	<(0.5, 0.7), (0.1, 0.2)>	<(0.4, 0.7), (0.1, 0.3)>	<(0.5, 0.7), (0.1, 0.4)>	<(0.2, 0.3), (0.1, 0.2)>	<(0.4, 0.6), (0.5, 0.8)>	<(0.2, 0.3), (0.6, 0.9)>
${C}_{5}$	<(0.3, 0.4), (0.4, 0.6)>	<(0.1, 0.4), (0.4, 0.6)>	<(0.1, 0.2), (0.3, 0.7)>	<(0.4, 0.6), (0.2, 0.3)>	<(0.3, 0.4), (0.3, 0.4)>	<(0.2, 0.6), (0.5, 0.6)>
${C}_{6}$	<(0.1, 0.4), (0.3, 0.6)>	<(0.2, 0.4), (0.3, 0.6)>	<(0.5, 0.6), (0.1, 0.3)>	<(0.2, 0.3), (0.1, 0.2)>	<(0.1, 0.4), (0.5, 0.7)>	<(0.2, 0.3), (0.4, 0.6)>
${C}_{7}$	<(0.2, 0.8), (0.1, 0.2)>	<(0.3, 0.7), (0.2, 0.3)>	<(0.6, 0.8), (0.1, 0.3)>	<(0.7, 0.8), (0.3, 0.4)>	<(0.3, 0.4), (0.5, 0.8)>	<(0.1, 0.2), (0.35, 0.4)>
${C}_{8}$	<(0.4, 0.7), (0.1, 0.3)>	<(0.2, 0.6), (0.2, 0.4)>	<(0.1, 0.8), (0.2, 0.3)>	<(0.5, 0.7), (0.3, 0.5)>	<(0.3, 0.4), (0.5, 0.6)>	<(0.1, 0.2), (0.4, 0.5)>
${C}_{9}$	<(0.2, 0.7), (0.2, 0.3)>	<(0.4, 0.7), (0.1, 0.3)>	<(0.6, 0.8), (0.2, 0.3)>	<(0.1, 0.3), (0.5, 0.6)>	<(0.5, 0.6), (0.7, 0.8)>	<(0.4, 0.5), (0.55, 0.6)>

**Table 7:** Sepsis characteristics on IVq-ROFS X_2_ by Expert P_2_.

	${{{A}}}_{1}$	${{{A}}}_{2}$	${{{A}}}_{3}$	${{{A}}}_{4}$	${{{A}}}_{5}$	${{{A}}}_{6}$
${C}_{1}$	<(0.3, 0.4), (0.5, 0.6)>	<(0.1, 0.3), (0.5, 0.7)>	<(0.2, 0.3), (0.3, 0.4)>	<(0.3, 0.5), (0.4, 0.5)>	<(0.4, 0.6), (0.7, 0.8)>	<(0.3, 0.4), (0.5, 0.6)>
${C}_{2}$	<(0.1, 0.3), (0.4, 0.5)>	<(0.1, 0.2, (0.4, 0.6)>	<(0.1, 0.3), (0.1, 0.5)>	<(0.3, 0.4), (0.2, 0.3)>	<(0.6, 0.7), (0.3, 0.4)>	<(0.1, 0.3), (0.5, 0.7)>
${C}_{3}$	<(0.4, 0.5), (0.5, 0.8)>	<(0.3, 0.5), (0.4, 0.5)>	<(0.5, 0.6), (0.6, 0.7)>	<(0.1, 0.2), (0.4, 0.6)>	<(0.3, 0.7), (0.4, 0.5)>	<(0.5, 0.6), (0.65, 0.7)>
${C}_{4}$	<(0.6, 0.7), (0.1, 0.2)>	<(0.6, 0.7), (0.1, 0.4)>	<(0.3, 0.4), (0.2, 0.4)>	<(0.2, 0.4), (0.4, 0.6)>	<(0.2, 0.6), (0.5, 0.7)>	<(0.4, 0.5), (0.5, 0.7)>
${C}_{5}$	<(0.2, 0.4), (0.5, 0.6)>	<(0.2, 0.4), (0.2, 0.6)>	<(0.2, 0.3), (0.3, 0.7)>	<(0.4, 0.6), (0.2, 0.3)>	<(0.7, 0.8), (0.3, 0.4)>	<(0.4, 0.6), (0.3, 0.4)>
${C}_{6}$	<(0.2, 0.3), (0.1, 0.6)>	<(0.3, 0.4), (0.2, 0.6)>	<(0.3, 0.6), (0.1, 0.3)>	<(0.1, 0.3), (0.6, 0.7)>	<(0.1, 0.2), (0.7, 0.9)>	<(0.3, 0.5), (0.6, 0.7)>
${C}_{7}$	<(0.1, 0.8), (0.2, 0.4)>	<(0.5, 0.7), (0.4, 0.5)>	<(0.6, 0.8), (0.1, 0.3)>	<(0.5, 0.6), (0.3, 0.4)>	<(0.2, 0.7), (0.3, 0.7)>	<(0.1, 0.2), (0.35, 0.4)>
${C}_{8}$	<(0.4, 0.6), (0.2, 0.3)>	<(0.5, 0.6), (0.1, 0.4)>	<(0.2, 0.5), (0.2, 0.3)>	<(0.5, 0.6), (0.1, 0.2)>	<(0.1, 0.3), (0.6, 0.7)>	<(0.5, 0.6), (0.5, 0.6)>
${C}_{9}$	<(0.3, 0.7), (0.1, 0.3)>	<(0.4, 0.5), (0.1, 0.2)>	<(0.6, 0.8), (0.2, 0.3)>	<(0.1, 0.2), (0.7, 0.8)>	<(0.4, 0.5), (0.6, 0.7)>	<(0.6, 0.7), (0.55, 0.6)>

Equations (13) and (15) are used for X_1_, X_2_ to obtain Tables 8, 9.

**Table 8:** Compromised MsAIVq-ROFS X_3_ based on IVq-ROFS X_1_, X_2_.

	${{{A}}}_{1}$	${{{A}}}_{2}$	${{{A}}}_{3}$	${{{A}}}_{4}$	${{{A}}}_{5}$	${{{A}}}_{6}$
${C}_{1}$	<(0.25, 0.4), (0.5, 0.6)>	<(0.1, 0.25), (0.55, 0.7)>	<(0.15, 0.25), (0.45, 0.6)>	<(0.35, 0.55), (0.3, 0.45)>	<(0.3, 0.6), (0.7, 0.8)>	<(0.2, 0.4), (0.6, 0.7)>
${C}_{2}$	<(0.15, 0.3), (0.4, 0.5)>	<(0.1, 0.25), (0.3, 0.6)>	<(0.1, 0.25), (0.1, 0.6)>	<(0.2, 0.3), (0.3, 0.4)>	<(0.5, 0.75), (0.2, 0.3)>	<(0.1, 0.35), (0.6, 0.75)>
${C}_{3}$	<(0.35, 0.45), (0.5, 0.8)>	<(0.2, 0.5), (0.45, 0.55)>	<(0.4, 0.6), (0.6, 0.7)>	<(0.15, 0.25), (0.45, 0.6)>	<(0.2, 0.6), (0.4, 0.6)>	<(0.4, 0.5), (0.65, 0.7)>
${C}_{4}$	<(0.55, 0.7), (0.1, 0.2)>	<(0.5, 0.7), (0.1, 0.35)>	<(0.4, 0.55), (0.15, 0.4)>	<(0.2, 0.35), (0.25, 0.4)>	<(0.3, 0.6), (0.5, 0.75)>	<(0.3, 0.4), (0.55, 0.8)>
${C}_{5}$	<(0.25, 0.4), (0.45, 0.6)>	<(0.15, 0.4), (0.3, 0.6)>	<(0.15, 0.25), (0.3, 0.7)>	<(0.4, 0.6), (0.2, 0.3)>	<(0.5, 0.6), (0.3, 0.4)>	<(0.3, 0.6), (0.4, 0.5)>
${C}_{6}$	<(0.15, 0.35), (0.2, 0.6)>	<(0.25, 0.4), (0.25, 0.6)>	<(0.4, 0.6), (0.1, 0.3)>	<(0.15, 0.3), (0.35, 0.45)>	<(0.1, 0.3), (0.6, 0.8)>	<(0.25, 0.4), (0.5, 0.65)>
${C}_{7}$	<(0.15, 0.8), (0.15, 0.3)>	<(0.4, 0.7), (0.3, 0.4)>	<(0.6, 0.8), (0.1, 0.3)>	<(0.6, 0.7), (0.3, 0.4)>	<(0.25, 0.55), (0.4, 0.75)>	<(0.1, 0.2), (0.35, 0.4)>
${C}_{8}$	<(0.4, 0.65), (0.15, 0.3)>	<(0.35, 0.6), (0.15, 0.4)>	<(0.15, 0.65), (0.2, 0.3)>	<(0.5, 0.65), (0.2, 0.35)>	<(0.2, 0.35), (0.55, 0.65)>	<(0.3, 0.4), (0.45, 0.6)>
${C}_{9}$	<(0.25, 0.7), (0.15, 0.3)>	<(0.4, 0.6), (0.1, 0.25)>	<(0.6, 0.8), (0.2, 0.3)>	<(0.1, 0.25), (0.6, 0.7)>	<(0.45, 0.55), (0.65, 0.75)>	<(0.5, 0.6), (0.55, 0.6)>

**Table 9:** Inclusive MsAIVq-ROFS X_4_ based on IVq-ROFSs X_1_, X_2_.

	${{{A}}}_{1}$	${{{A}}}_{2}$	${{{A}}}_{3}$	${{{A}}}_{4}$	${{{A}}}_{5}$	${{{A}}}_{6}$
${C}_{1}$	<(0.2, 0.4), (0.5, 0.6)>	<(0.1, 0.3), (0.5, 0.7)>	<(0.1, 0.3), (0.3, 0.8)>	<(0.3, 0.6), (0.2, 0.5)>	<(0.2, 0.6), (0.7, 0.8)>	<(0.1, 0.4), (0.5, 0.8)>
${C}_{2}$	<(0.1, 0.3), (0.4, 0.5)>	<(0.1, 0.3), (0.2, 0.6)>	<(0.1, 0.3), (0.1, 0.7)>	<(0.1, 0.4), (0.2, 0.5)>	<(0.4, 0.8), (0.1, 0.4)>	<(0.1, 0.4), (0.5, 0.8)>
${C}_{3}$	<(0.3, 0.5), (0.5, 0.8)>	<(0.2, 0.5), (0.4, 0.6)>	<(0.3, 0.6), (0.6, 0.7)>	<(0.1, 0.3), (0.4, 0.6)>	<(0.1, 0.7), (0.4, 0.7)>	<(0.3, 0.6), (0.65, 0.7)>
${C}_{4}$	<(0.5, 0.7), (0.1, 0.2)>	<(0.4, 0.7), (0.1, 0.4)>	<(0.3, 0.7), (0.1, 0.4)>	<(0.2, 0.4), (0.1, 0.6)>	<(0.2, 0.6), (0.5, 0.8)>	<(0.2, 0.5), (0.5, 0.8)>
${C}_{5}$	<(0.2, 0.4), (0.4, 0.6)>	<(0.1, 0.4), (0.2, 0.6)>	<(0.1, 0.3), (0.3, 0.7)>	<(0.4, 0.6), (0.2, 0.3)>	<(0.3, 0.8), (0.3, 0.4)>	<(0.2, 0.6), (0.3, 0.6)>
${C}_{6}$	<(0.1, 0.4), (0.1, 0.6)>	<(0.2, 0.4), (0.2, 0.6)>	<(0.3, 0.6), (0.1, 0.3)>	<(0.1, 0.3), (0.1, 0.7)>	<(0.1, 0.4), (0.5, 0.9)>	<(0.2, 0.5), (0.4, 0.7)>
${C}_{7}$	<(0.1, 0.8), (0.1, 0.4)>	<(0.3, 0.7), (0.2, 0.5)>	<(0.6, 0.8), (0.1, 0.3)>	<(0.5, 0.8), (0.3, 0.4)>	<(0.2, 0.7), (0.3, 0.7)>	<(0.1, 0.2), (0.35, 0.4)>
${C}_{8}$	<(0.4, 0.7), (0.1, 0.3)>	<(0.2, 0.6), (0.1, 0.4)>	<(0.1, 0.8), (0.2, 0.3)>	<(0.5, 0.7), (0.1, 0.5)>	<(0.1, 0.4), (0.5, 0.7)>	<(0.1, 0.6), (0.4, 0.6)>
${C}_{9}$	<(0.2, 0.7), (0.1, 0.3)>	<(0.4, 0.7), (0.1, 0.3)>	<(0.6, 0.8), (0.2, 0.3)>	<(0.1, 0.3), (0.5, 0.8)>	<(0.4, 0.6), (0.6, 0.8)>	<(0.4, 0.7), (0.55, 0.6)>

The processing steps of proposed improved CoCoSo method are as follows:

*Step 1* Firstly, IVq-ROFSs X_1_ and X_2_ obtained from P_1_ and P_2_ for one patient are aggregated as X_3_ and X_4_ which are obtained by the two proposed aggregation methods, as shown in Tables 8 and 9, respectively.

*Step 2* From Table 5, we know that C_1_, C_4_, C_6_, C_7_, C_8_ are benefit criteria, C_2_, C_3_, C_5_, C_9_ are cost criteria. So according to Eqs. (19) and (20), the aggregated set of patient attributes is normalized. Here we take X_3_ obtained by inclusive MsAIVq-ROFS as an example, we can get the normalized decision matrix as Table 10.

**Table 10:** The normalized decision matrix based on inclusive MsAIVq-ROFS.

	${{{A}}}_{1}$	${{{A}}}_{2}$	${{{A}}}_{3}$	${{{A}}}_{4}$	${{{A}}}_{5}$	${{{A}}}_{6}$
${C}_{1}$	0.182420	0.153717	0.159079	0.174640	0.159815	0.1703290
${C}_{2}$	0.155580	0.165814	0.181164	0.182923	0.162936	0.1515831
${C}_{3}$	0.214251	0.157638	0.112250	0.216447	0.152269	0.1471450
${C}_{4}$	0.160550	0.164578	0.176102	0.141195	0.190311	0.1672634
${C}_{5}$	0.145770	0.182718	0.237836	0.178478	0.127599	0.1275994
${C}_{6}$	0.160046	0.173863	0.170409	0.152562	0.151986	0.1911341
${C}_{7}$	0.120456	0.182259	0.159961	0.203587	0.188439	0.1452982
${C}_{8}$	0.154684	0.167102	0.138998	0.172985	0.179847	0.1863834
${C}_{9}$	0.241146	0.195087	0.213716	0.190993	0.117912	0.0411464

*Step 3* Calculate the objective weights of the attributes by using the Eqs. (21–22), and the corresponding weights of each attribute are shown as the following Table 11.

**Table 11:** Objective weighting of attributes.

Attributes	Weights
${C}_{1}$	0.11373375
${C}_{2}$	0.10907751
${C}_{3}$	0.10671905
${C}_{4}$	0.11856914
${C}_{5}$	0.10717358
${C}_{6}$	0.11235538
${C}_{7}$	0.11573375
${C}_{8}$	0.11367079
${C}_{9}$	0.10480883

*Step 4* According to the compromise calculation steps of the IVq-ROFS-CoCoSo method, the ranking algorithm of the alternatives is carried out sequentially, and the arithmetic weighted sum and arithmetic weighted product of each alternative are first calculated by the Eqs. (23, 24) as shown as Table 12.

**Table 12:** Arithmetic weighted sum and arithmetic weighted product of each alternative.

	${\overline{{{S}}} }_{{{\omega}}{{i}}}$	${\overline{{{M}}} }_{{{\omega}}{{i}}}$
${{{A}}}_{1}$	0.17002150	7.37434996
${{{A}}}_{2}$	0.17155811	7.39336800
${{{A}}}_{3}$	0.17206130	7.38270240
${{{A}}}_{4}$	0.17916780	7.42561249
${{{A}}}_{5}$	0.16012616	7.32930866
${{{A}}}_{6}$	0.14890692	7.22983220

Next, the values of the three types of compromise functions are calculated for each alternative by the Eq. (25) as shown as Table 13, where $\varepsilon =0.5$.

**Table 13:** The values of the three types of compromise functions.

	${\overline{S} }_{i}^{1}$	${\overline{S} }_{i}^{2}$	${\overline{S} }_{i}^{3}$
${{{A}}}_{1}$	0.167143782	2.161786281	0.992056466
${{{A}}}_{2}$	0.167599165	2.174736032	0.994759325
${{{A}}}_{3}$	0.167374019	2.176640035	0.993423006
${{{A}}}_{4}$	0.168482125	2.230299637	1
${{{A}}}_{5}$	0.165926673	2.089103153	0.984832504
${{{A}}}_{6}$	0.163474236	2	0.970276436

Finally, the alternatives are ranked by the improved score function Eq. (26). The result is as shown as Table 14.

**Table 14:** Score values and sort results based on compromised MsAIVq-ROFS.

	${{{A}}}_{1}$	${{{A}}}_{2}$	${{{A}}}_{3}$	${{{A}}}_{4}$	${{{A}}}_{5}$	${{{A}}}_{6}$	Ranking of the alternatives
Score	1.670855	1.674434	1.673780	1.685323	1.655935	1.633128	${{{A}}}_{4}>{{{A}}}_{2}>{{{A}}}_{3}>{{{A}}}_{1}>{{{A}}}_{5}>{{{A}}}_{6}$

Similar to the above process, the final scores of the inclusive MsAIVq-ROFS based on X_1,_ X_2_ and the ranking results of the alternatives are shown in Table 15.

**Table 15:** Score values and sort results based on inclusive MsAIVq-ROFS.

	${{{A}}}_{1}$	${{{A}}}_{2}$	${{{A}}}_{3}$	${{{A}}}_{4}$	${{{A}}}_{5}$	${{{A}}}_{6}$	Ranking of the alternatives
Score	1.100536	1.110470	1.085934	1.008798	1.001094	0.961254	${{{A}}}_{2}>{{{A}}}_{1}>{{{A}}}_{3}>{{{A}}}_{5}>{{{A}}}_{4}>{{{A}}}_{6}$

From the ranking results obtained by the proposed method for the compromised MsAIVq-ROFS and the inclusive MsAIVq-ROFS based on two identical IVq-ROFSs, it can be seen that the two different multi-source aggregation strategies can give the decision maker an optimistic or pessimistic opinion on the decision.

## Sensitivity analysis and comparative analysis

In this section, we first make sensitivity analysis. Then we made the comparative analysis with other methods to verify the feasibility of our method. Finally, we describe the merits of our method.

### Sensitivity analysis

**Figure 3:**
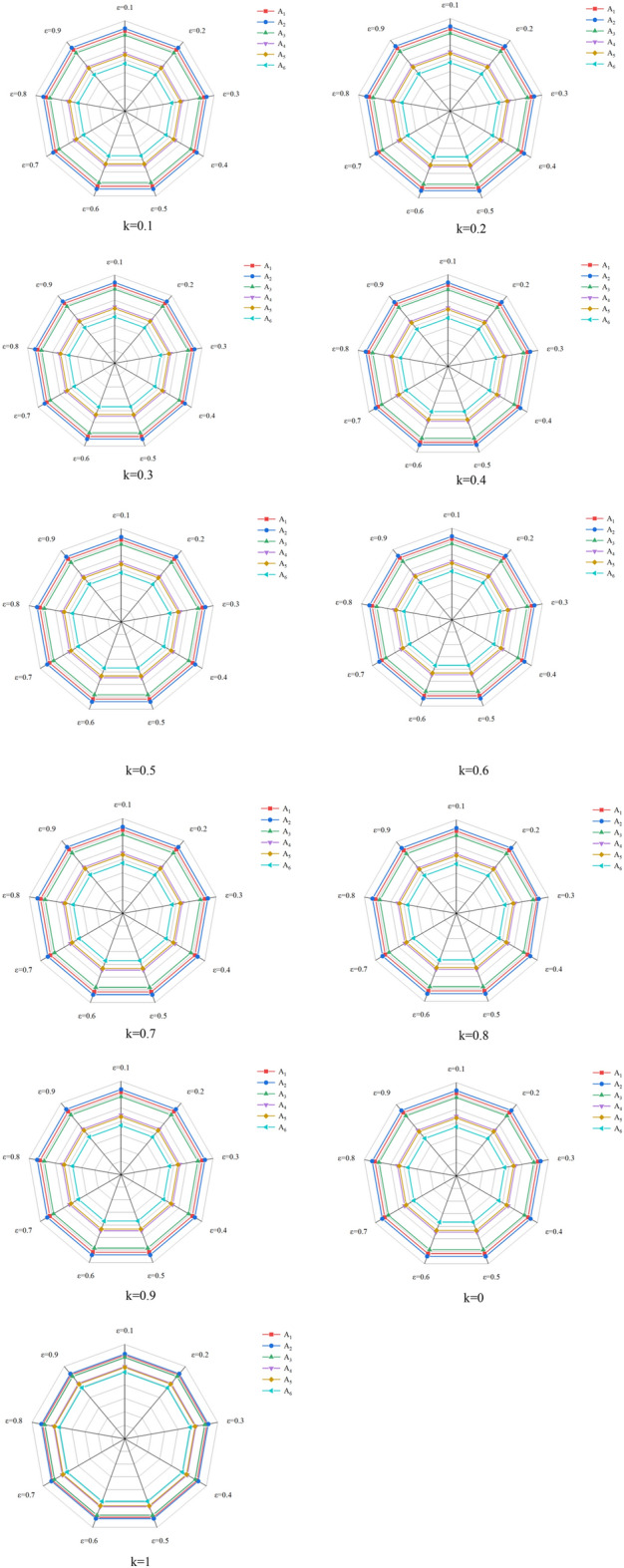
Sensitivity analysis for parameter changes.

This section focuses on the sensitivity analysis of the two parameters of the proposed method. The first parameter is the k in the IVq-ROFS-based attribute weight calculation method Eq. (21), and the second parameter is the equilibrium parameter $\varepsilon$ in the Eq. (25). So, based on the inclusive MsAIVq-ROFS in the previous section data, the sensitivity analysis is performed for the two parameters set in increments of 0.1 from 0. The results obtained from the analysis are shown below in Fig. 3.

The figure shows that for different values of k, the balance parameter does not affect the ranking for any value. However, different values of k will have a particular impact on the difficulty of ranking the alternatives. It can be clearly seen in the above figure that when k = 1, the gap between the scores of A_1_, A_2_ and A_3_ and between the scores of A_4_ and A_5_ is not clear. For other values of k, alternatives can be ranked more clearly.

### Comparison analysis with Other MAGDM

**Figure 4:**
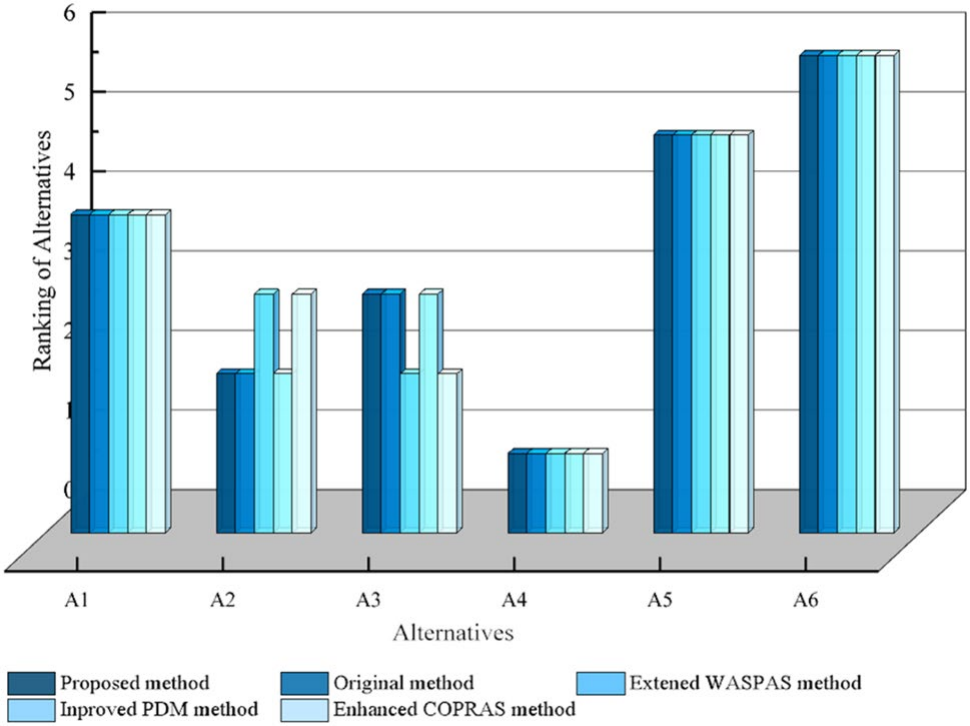
Results analysis with other methods.

Based on Table 8 in “The decision process of Sepsis diagnosis” section, we rank the alternatives using the traditional CoCoSo method^58^, the extended WASPAS method proposed by^56^, the improved PDM method proposed in^57^, and the COPRAS method proposed in^52^. After using the weights obtained from the weighting method proposed in this paper, the process of the rest of the steps is basically the same, and the final ranking of the alternatives is calculated. The calculations results are shown as Table 16 and Fig. 4 below.

**Table 16:** Results analysis with other methods.

	${{{A}}}_{1}$	${{{A}}}_{2}$	${{{A}}}_{3}$	${{{A}}}_{4}$	${{{A}}}_{5}$	${{{A}}}_{6}$	Ranking of the alternatives
Proposed method	0.921517	0.938470	0.932458	0.979045	0.867999	0.776336	${{{A}}}_{4}>{{{A}}}_{2}>{{{A}}}_{3}>{{{A}}}_{1}>{{{A}}}_{5}>{{{A}}}_{6}$
Original CoCoSo method^58^	1.276959	1.303964	1.294251	1.369873	1.194671	1.061908	${{{A}}}_{4}>{{{A}}}_{2}>{{{A}}}_{3}>{{{A}}}_{1}>{{{A}}}_{5}>{{{A}}}_{6}$
WASPAS method in^56^	0.342676	0.343585	0.344032	0.355813	0.314802	0.275809	${{{A}}}_{4}>{{{A}}}_{3}>{{{A}}}_{2}>{{{A}}}_{1}>{{{A}}}_{5}>{{{A}}}_{6}$
PDM method in^57^	0.243821	0.255627	0.252879	0.268796	0.235792	0.212178	${{{A}}}_{4}>{{{A}}}_{2}>{{{A}}}_{3}>{{{A}}}_{1}>{{{A}}}_{5}>{{{A}}}_{6}$
COPRAS method in^52^	0.843826	0.883425	0.982574	1.286425	0.683835	0.528759	${{{A}}}_{4}>{{{A}}}_{3}>{{{A}}}_{2}>{{{A}}}_{1}>{{{A}}}_{5}>{{{A}}}_{6}$

From the Table 16 and Fig. 4, it can be seen that this paper's method can obtain consistent optimal solutions with other methods in the same data set, and the ranking of alternatives are almost the same, which indicates the correctness of this paper's method.

### Advantages analysis

In this section, we compare the proposed method with the existing methods to discuss the advantages of our method.

1. Aggregation method

In this paper, we propose two new multi-source aggregation methods in a more straightforward form as a pre-data processing method for MAGDM. The method in^56^ used the additive mean of fuzzy numbers to aggregate fuzzy data, and in^55^ the Archimedean Muirhead mean operators were used to aggregate fuzzy data. In other studies, the complex aggregation operators^62–64^ were applied, which are complicated to implement and not easy to understand. Compared with them, the proposed aggregation method is easier to understand and implement, and the proposed aggregation method can be extended into most of MAGDM.

(2) Weight Calculation method.

A novel fuzzy entropy with a parameter based on IVq-ROFS, which is more flexible and versatile due to the settable parameter, is proposed. The entropy weight method is a known proven weight calculation method with excellent performance, and fuzzy entropy with a parameter is combined with the traditional entropy weight method, and can be applied to IVq-ROFS. Scholars have yet to apply the entropy weight method to IVq-ROFS in previous studies, and this paper fills the gap in this area. Compared with the method in^55,56,58^, the more objective attribute weights will be obtained in the method proposed.

(3) Enhanced compromise score in CoCoSo method.

In the original CoCoSo method, there is an unavoidable problem, as shown in Eq. (9) and (10), in which $0\le {S}_{i}^{1}\le 1$, $0\le {S}_{i}^{3}\le 1$, ${S}_{i}^{2}\ge 2$, the value domains of the three dependent compromise scores are different, directly affecting the final score calculation. It is necessary to convert three dependent compromise scores to a unified scale for the CoCoSo method. So this paper uses the logarithmic function to limit the value domain of ${S}_{i}^{2}$ to $[\mathrm{0,1}]$ which can eliminate dimensional differences among three dependent compromise scores of the existing CoCoSo methods.

(4) Higher versatility and scalability.

The proposed approach has excellent versatility and scalability, which is not only limited to the IVq-ROFS, but also applies to IVIFS, IVPFS and IVFFS.

We summarize the merits of our method in Table 17.

**Table 17:** The comparison of existing methods and the proposed method.

Approach	Weight calculation	Information measure	Aggregation method	Eliminating dimensional differences for three compromise scores	Alternative evaluations	Scalability	Benchmarks
Proposed method	Most objective	Fuzzy entropy with a parameter	Easiest to understand and implement	Yes	IVq-ROFS	higher	Fuzzy entropy and improved CoCoSo method
Original CoCoSo method^58^	Subjective	–	–	No	Exact value	lower	CoCoSo method
Method in^56^	More objective	Similarity mesaure	–	–	IVPFS	lower	Similarity measures and improved score function
Method in^55^	Subjective	–	Hardest to understand and implement	–	IVq-ROFS	higher	Archimedean Muirhead mean operators
Method in^57^	Subjective	Possibility degree measure	Easier to understand and implement	–	IVq-ROFS	higher	Improved PDM method
Method in^52^	More objective	–	Harder to understand and implement	–	IVq-ROFS	higher	Enhanced COPRAS method

## Conclusion

In this study, a novel MAGDM method based on IVq-ROFS is proposed. Firstly, a new multi-source IVq-ROFS aggregation method is proposed, which is simpler and easier to implement than the existing complex aggregation methods. Secondly, a parametric fuzzy entropy based on IVq-ROFS, which is more flexible and universal than the traditional fuzzy entropy, is introduced. Based on the fuzzy entropy, we propose a fuzzy entropy weighting method to determine the objective weights of attributes. After that, we improve the defects of the original CoCoSo method by restricting the three compromise scores to the same range. It solves the problem of dimensional differences in the original one. Finally, a novel improved CoCoSo method based on IVq-ROFS is proposed. It is created based on these theoretical innovations and applied to sepsis case analysis.

Among the main implications of this study for sepsis diagnosis are: first, because early sepsis is difficult to be diagnosed exactly by doctors, this problem can be solved by using fuzzy theory. Secondly, CoCoSo, as a multi-attribute decision model, can synthesize the diagnoses of multiple experts to reduce the problem of misdiagnosis in the early stage of sepsis. Finally, doctors nowadays use precise instruments for medical diagnosis, however, the early symptoms of sepsis make doctors uncertain even with precise instruments, CoCoSo in a fuzzy environment allows multiple doctors to consider the summed assessment of multiple experts from a fuzzy point of view in the early stages of sepsis, which helps to minimize the risk of disease for the patient.

Some outstanding issues require additional investigation in future research. The MAGDM method in this study is proposed based on IVq-ROFS and does not consider hesitant fuzzy information and complex fuzzy information. In later work, more diverse fuzzy information will be considered. The application of the study focuses on sepsis diagnosis, and future work will try to apply it to psychological diagnosis or supply chain location.

## Data Availability

The datasets generated and/or analyzed during the current study are available to gain in this paper.

## References

[1] Qin H, Fei Q, Ma X, Chen W (2021). A new parameter reduction algorithm for soft sets based on chi-square test. Appl. Intell..

[2] Ma X, Fei Q, Qin H, Zhou X, Li H (2019). New improved normal parameter reduction method for fuzzy soft set. IEEE Access.

[3] Abid M, Saqlain M (2023). Utilizing edge cloud computing and deep learning for enhanced risk assessment in China’s international trade and investment. Int. J. Knowl. Innov. Stud..

[4] Zadeh L (1965). Fuzzy sets. Inf. Control.

[5] Takeuti G, Titani S (1984). Intuitionistic fuzzy logic and intuitionistic fuzzy set theory. J. Symb. Logic.

[6] Verma R, Sharma B (2015). Intuitionistic fuzzy Einstein prioritized weighted average operators and their application to multiple attribute group decision making. Appl. Math. Inf. Sci..

[7] Garg H (2016). Generalized intuitionistic fuzzy interactive geometric interaction operators using Einstein t-norm and t-conorm and their application to decision making. Comput. Ind. Eng..

[8] Chaudhuri A (2015). Intuitionistic fuzzy possibilistic c means clustering algorithms. Adv. Fuzzy Syst..

[9] Ezghari S, Zahi A, Zenkouar K (2017). A new nearest neighbor classification method based on fuzzy set theory and aggregation operators. Expert Syst. Appl..

[10] Verma H, Agrawal R, Sharan A (2016). An improved intuitionistic fuzzy c-means clustering algorithm incorporating local information for brain image segmentation. Appl. Soft Comput..

[11] Ananthi V, Balasubramaniam P, Kalaiselvi T (2016). A new fuzzy clustering algorithm for the segmentation of brain tumor. Soft Comput..

[12] Boran FE, Akay D (2014). A biparametric similarity measure on intuitionistic fuzzy sets with applications to pattern recognition. Inf. Sci..

[13] Hwang C-M, Yang M-S, Hung W-L, Lee M-G (2012). A similarity measure of intuitionistic fuzzy sets based on the Sugeno integral with its application to pattern recognition. Inf. Sci..

[14] Turksen IB (1986). Interval valued fuzzy sets based on normal forms. Fuzzy Sets Syst..

[15] Atanassov, K. T. and Atanassov, K. T. Interval valued intuitionistic fuzzy sets. In *Intuitionistic Fuzzy Sets: Theory and Applications* 139–177 (1999).

[16] Yager RR (2013). Pythagorean membership grades in multicriteria decision making. IEEE Trans. Fuzzy Syst..

[17] Ma Z, Xu Z (2016). Symmetric Pythagorean fuzzy weighted geometric/averaging operators and their application in multicriteria decision-making problems. Int. J. Intell. Syst..

[18] Zhang X (2016). Multicriteria Pythagorean fuzzy decision analysis: A hierarchical QUALIFLEX approach with the closeness index-based ranking methods. Inf. Sci..

[19] Senapati T, Yager RR (2020). Fermatean fuzzy sets. J. Ambient Intell. Hum. Comput..

[20] Khan A, Wang L (2023). Generalized and group-generalized parameter based Fermatean fuzzy aggregation operators with application to decision-making. Int J. Knowl. Innov. Stud..

[21] Jeevaraj S (2021). Ordering of interval-valued Fermatean fuzzy sets and its applications. Expert Syst. Appl..

[22] Joshi BP, Singh A, Bhatt PK, Vaisla KS (2018). Interval valued q-rung orthopair fuzzy sets and their properties. J. Intell. Fuzzy Syst..

[23] Yager RR (2016). Generalized orthopair fuzzy sets. IEEE Trans. Fuzzy Syst..

[24] Farid HMA, Riaz M (2023). q-rung orthopair fuzzy Aczel-Alsina aggregation operators with multi-criteria decision-making. Eng. Appl. Artif. Intell..

[25] Riaz M, Farid HMA, Shakeel HM, Aslam M, Mohamed SH (2021). Innovative q-rung orthopair fuzzy prioritized aggregation operators based on priority degrees with application to sustainable energy planning: A case study of Gwadar. AIMS Math..

[26] Hussain A, Latif S, Ullah K (2022). A novel approach of picture fuzzy sets with unknown degree of weights based on Schweizer-Sklar aggregation operators. J. Innov. Res. Math. Comput. Sci..

[27] Khan Q, Jabeen K (2022). Schweizer-Sklar aggregation operators with unknown weight for picture fuzzy information. J. Innov. Res. Math. Comput. Sci..

[28] Riaz M, Farid H (2023). Enhancing green supply chain efficiency through linear Diophantine fuzzy soft-max aggregation operators. J. Ind. Intell..

[29] Rehman UU, Mahmood T (2021). Picture fuzzy N-soft sets and their applications in decision-making problems. Fuzzy Inf. Eng..

[30] Ali Z, Mahmood T, Yang M-S (2020). TOPSIS method based on complex spherical fuzzy sets with Bonferroni mean operators. Mathematics.

[31] Mahmood T, Ur Rehman U (2022). A novel approach towards bipolar complex fuzzy sets and their applications in generalized similarity measures. Int. J. Intell. Syst..

[32] Zulqarnain RM, Ali R, Awrejcewicz J, Siddique I, Jarad F, Iampan A (2022). Some Einstein geometric aggregation operators for Q-rung orthopair fuzzy soft set with their application in MCDM. IEEE Access.

[33] Zulqarnain RM, Rehman HKU, Awrejcewicz J, Ali R, Siddique I, Jarad F, Iampan A (2022). Extension of Einstein average aggregation operators to medical diagnostic approach under Q-rung orthopair fuzzy soft set. IEEE Access.

[34] Zulqarnain RM, Siddique I, EI-Morsy S (2022). Einstein-ordered weighted geometric operator for Pythagorean fuzzy soft set with its application to solve MAGDM problem. Math. Probl. Eng..

[35] Zulqarnain RM, Siddique I, Iampan A, Awrejcewicz J, Bednarek M, Ali R, Asif M (2022). Novel multicriteria decision making approach for interactive aggregation operators of q-rung orthopair fuzzy soft set. IEEE Access.

[36] Zulqarnain RM, Siddique I, Jarad F, Hamed Y, Abualnaja KM, Iampan A (2022). Einstein aggregation operators for Pythagorean fuzzy soft sets with their application in multiattribute group decision-making. J. Funct. Spaces.

[37] Zulqarnain RM, Garg H, Ma W-X, Siddique I (2024). Optimal cloud service provider selection: An MADM framework on correlation-based TOPSIS with interval-valued q-rung orthopair fuzzy soft set. Eng. Appl. Artif. Intell..

[38] Garg H, Ali Z, Mahmood T (2021). Algorithms for complex interval-valued q-rung orthopair fuzzy sets in decision making based on aggregation operators, AHP, and TOPSIS. Expert Syst..

[39] Mahmood T, Ali Z (2023). Analysis of Maclaurin symmetric mean operators for managing complex interval-valued q-Rung orthopair fuzzy setting and their applications. J. Comput. Cognit. Eng..

[40] Dong X, Ali Z, Mahmood T, Liu P (2023). Yager aggregation operators based on complex interval-valued q-rung orthopair fuzzy information and their application in decision making. Complex Intell. Syst..

[41] Verma R (2020). Multiple attribute group decision-making based on order-α divergence and entropy measures under q-rung orthopair fuzzy environment. Int. J. Intell. Syst..

[42] Arya V, Kumar S (2021). Extended TODIM method based on VIKOR for q-rung orthopair fuzzy information measures and their application in MAGDM problem of medical consumption products. Int. J. Intell. Syst..

[43] Liu L, Cao W, Shi B, Tang M (2019). Large-scale green supplier selection approach under a q-rung interval-valued orthopair fuzzy environment. Processes.

[44] Zhang G, Yuan G (2023). Generalized interval-valued q-Rung orthopair hesitant fuzzy choquet operators and their application. Symmetry.

[45] Nasir A, Jan N, Pamucar D, Khan SU (2023). Analysis of cybercrimes and security in FinTech industries using the novel concepts of interval-valued complex q-rung orthopair fuzzy relations. Expert Syst. Appl..

[46] Qi X, Ali Z, Mahmood T, Liu P (2023). Multi-attribute decision-making method based on complex interval-valued q-Rung orthopair linguistic Heronian mean operators and their application. Int. J. Fuzzy Syst..

[47] Giri BK, Roy SK, Deveci M (2023). Projection based regret theory on three-way decision model in probabilistic interval-valued q-rung orthopair hesitant fuzzy set and its application to medicine company. Artif. Intell. Rev..

[48] Ahemad F, Khan AZ, Mehlawat MK, Gupta P, Roy SK (2023). Multi-attribute group decision-making for solid waste management using interval-valued q-rung orthopair fuzzy COPRAS. RAIRO-Oper. Res..

[49] Ahemad F, Mehlawat MK, Gupta P (2023). A GRA approach to a MAGDM problem with interval-valued q-rung orthopair fuzzy information. Soft Comput..

[50] Garg H (2021). New exponential operation laws and operators for interval-valued q-rung orthopair fuzzy sets in group decision making process. Neural Comput. Appl..

[51] Jin C, Ran Y, Zhang G (2021). Interval-valued q-rung orthopair fuzzy FMEA application to improve risk evaluation process of tool changing manipulator. Appl. Soft Comput..

[52] Seker S, Bağlan FB, Aydin N, Deveci M, Ding W (2023). Risk assessment approach for analyzing risk factors to overcome pandemic using interval-valued q-rung orthopair fuzzy decision making method. Appl. Soft Comput..

[53] Yang Z, Zhang L, Li T (2021). Group decision making with incomplete interval-valued q-rung orthopair fuzzy preference relations. Int. J. Intell. Syst..

[54] Gurmani SH, Garg H, Zulqarnain RM, Siddique I (2023). Selection of unmanned aerial vehicles for precision agriculture using interval-valued q-Rung orthopair fuzzy information based TOPSIS method. Int. J. Fuzzy Syst..

[55] Gao H, Ju Y, Zhang W, Ju D (2019). Multi-attribute decision-making method based on interval-valued *q*-rung orthopair fuzzy Archimedean muirhead mean operators. IEEE Access.

[56] Al-Barakati A, Mishra AR, Mardani A, Rani P (2022). An extended interval-valued Pythagorean fuzzy WASPAS method based on new similarity measures to evaluate the renewable energy sources. Appl. Soft Comput..

[57] Garg H (2021). A new possibility degree measure for interval-valued q-rung orthopair fuzzy sets in decision-making. Int. J. Intell. Syst..

[58] Yazdani M, Zarate P, Kazimieras Zavadskas E, Turskis Z (2019). A combined compromise solution (CoCoSo) method for multi-criteria decision-making problems. Manag. Decis..

[59] Hung WL, Yang MS (2006). Fuzzy entropy on intuitionistic fuzzy sets. Int. J. Intell. Syst..

[60] Zhang Q-S, Jiang S, Jia B, Luo S (2010). Some information measures for interval-valued intuitionistic fuzzy sets. Inf. Sci..

[61] Petry FE, Yager RR (2022). Interval-valued fuzzy sets aggregation and evaluation approaches. Appl. Soft Comput..

[62] Yang Z, Li X, Cao Z, Li J (2019). Q-rung orthopair normal fuzzy aggregation operators and their application in multi-attribute decision-making. Mathematics.

[63] Wang J, Wei G, Wang R, Alsaadi FE, Hayat T, Wei C, Zhang Y, Wu J (2019). Some q-rung interval-valued orthopair fuzzy Maclaurin symmetric mean operators and their applications to multiple attribute group decision making. Int. J. Intell. Syst..

[64] Donyatalab, Y., Farrokhizadeh, E., Shishavan, S. A. S. and Seifi, S. H. *Hamacher aggregation operators based on interval-valued q-rung orthopair fuzzy sets and their applications to decision making problems*. Springer, City, 2020.

[65] Jozwiak M, Monnet X, Teboul JL (2016). Implementing sepsis bundles. Ann. Transl. Med..

[66] Wang, X. and He, Y. Sepsis Prediction with Temporal Convolutional Networks. arXiv preprint arXiv:2205.15492 (2022).

[67] O’Brien JM, Ali NA, Aberegg SK, Abraham E (2007). Sepsis. Am. J. Med..

[68] Pierrakos C, Vincent J-L (2010). Sepsis biomarkers: A review. Crit. Care.

[69] Hotchkiss RS, Karl IE (2003). The pathophysiology and treatment of sepsis. N. Engl. J. Med..

[70] Medzhitov R (2008). Origin and physiological roles of inflammation. Nature.

[71] Ahmed AU (2011). An overview of inflammation: Mechanism and consequences. Front. Biol..

